# A Novel Magnetic Responsive miR‐26a@SPIONs‐OECs for Spinal Cord Injury: Triggering Neural Regeneration Program and Orienting Axon Guidance in Inhibitory Astrocytic Environment

**DOI:** 10.1002/advs.202304487

**Published:** 2023-10-03

**Authors:** Xue Gao, Shengyou Li, Yujie Yang, Shijie Yang, Beibei Yu, Zhijie Zhu, Teng Ma, Yi Zheng, Bin Wei, Yiming Hao, Haining Wu, Yongfeng Zhang, Lingli Guo, Xueli Gao, Yitao Wei, Borui Xue, Jianzhong Li, Xue Feng, Lei Lu, Bing Xia, Jinghui Huang

**Affiliations:** ^1^ Department of Orthopaedics Xijing Hospital Fourth Military Medical University Xi'an 710032 P. R. China; ^2^ Department of Neurosurgery The Second Affiliated Hospital of Xi'an Jiao Tong University Xi'an 710032 P. R. China; ^3^ School of Ecology and Environment Northwestern Polytechnical University Xi'an 710072 P. R. China; ^4^ Department of Thoracic Surgery The Second Affiliated Hospital of Xi'an Jiao Tong University Xi'an 710032 P. R. China; ^5^ Department of Cell Biology School of Medicine Northwest University Xi'an 710032 P. R. China; ^6^ State Key Laboratory of Military Stomatology National Clinical Research Center for Oral Diseases Shaanxi International Joint Research Center for Oral Diseases Department of Oral Anatomy and Physiology and TMD School of Stomatology the Fourth Military Medical University Xi'an 710032 P. R. China

**Keywords:** cell orientation, miR‐26a, olfactory ensheathing cell, spinal cord injury, SPIONs

## Abstract

Addressing the challenge of promoting directional axonal regeneration in a hostile astrocytic scar, which often impedes recovery following spinal cord injury (SCI), remains a daunting task. Cell transplantation is a promising strategy to facilitate nerve restoration in SCI. In this research, a pro‐regeneration system is developed, namely miR‐26a@SPIONs‐OECs, for olfactory ensheathing cells (OECs), a preferred choice for promoting nerve regeneration in SCI patients. These entities show high responsiveness to external magnetic fields (MF), leading to synergistic multimodal cues to enhance nerve regeneration. First, an MF stimulates miR‐26a@SPIONs‐OECs to release extracellular vesicles (EVs) rich in miR‐26a. This encourages axon growth by inhibiting PTEN and GSK‐3β signaling pathways in neurons. Second, miR‐26a@SPIONs‐OECs exhibit a tendency to migrate and orientate along the direction of the MF, thereby potentially facilitating neuronal reconnection through directional neurite elongation. Third, miR‐26a‐enriched EVs from miR‐26a@SPIONs‐OECs can interact with host astrocytes, thereby diminishing inhibitory cues for neurite growth. In a rat model of SCI, the miR‐26a@SPIONs‐OECs system led to significantly improved morphological and motor function recovery. In summary, the miR‐26a@SPIONS‐OECs pro‐regeneration system offers innovative insights into engineering exogenous cells with multiple additional cues, augmenting their efficacy for stimulating and guiding nerve regeneration within a hostile astrocytic scar in SCI.

## Graphical abstract

A pro‐regeneration system, namely miR‐26a@SPIONs‐OECs, is developed to release extracellular vesicles enrich with miR‐26a under external magnetic field stimulation, promoting axon growth and directional neurite elongation, inhibiting the activity of host astrocytes, and leading to a significantly improved morphological and motor function recovery in a rat model of spinal cord injury.

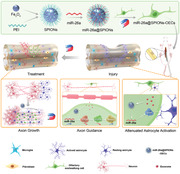

## 1 Introduction

Spinal cord injury (SCI) is one of the most catastrophic medical conditions, involving intricate pathological states that often result in a varying range of permanent neurological impairments.^[^ ^1^ ^]^ Globally, about half a million cases of traumatic SCI presenting with motor and/or sensory function impairments are reported each year.^[^ ^2^ ^]^ Despite considerable progress in SCI research, an established cure for SCI remains elusive. Central nervous system (CNS) neurons exhibit a limited ability to regenerate axons after trauma, unlike those of the peripheral nervous system (PNS).^[^ ^3^ ^]^ Further, the sporadic and disorganized growth of newly regenerated axons compromises the effectiveness of repair in SCI.^[^ ^4^ ^]^ Host astrocytes around the lesion proliferate and intertwine their extended protrusions to form a glial scar, creating an inhibitory environment for neurite growth.^[^ ^5^ ^]^ As a result, the constrained regenerative capacity of damaged neurons and the physical barriers created by glial scar tissue significantly impede nerve regeneration and functional restoration following SCI.

The concept of cell transplantation presents a promising strategy for facilitating nerve restoration after SCI.^[^ ^6^ ^]^ Exogenous cells contribute to the partial enhancement of functional restoration after SCI through mechanisms such as neuroprotection, immunomodulation, axon sprouting and/or regeneration, neuronal relay creation, and myelin regeneration.^[^ ^7^ ^]^ Particularly in the CNS, transplantation of olfactory ensheathing cells (OECs) has emerged as an appealing option for neural repair due to their potential to support axon migration from the PNS to the CNS throughout life.^[^ ^8^ ^]^ The use of OECs in SCI treatment has recently been extensively investigated.^[^ ^9^ ^]^ OECs protect lesioned areas by mitigating the inflammatory response and reducing cavitation.^[^ ^10^ ^]^ Furthermore, various studies have demonstrated that OECs augment nerve repair following SCI by promoting remyelination, angiogenesis, and axonal regrowth/plasticity across different tracts.^[^ ^9^, ^11^ ^]^ However, transplanted OECs are often isolated and surrounded by activated host astrocytes, severely limiting their migratory ability and bioactivity, thereby drastically reducing the regenerative competency of the exogenous OECs.^[^ ^12^ ^]^ Additionally, the irregular distribution of OECs within the lesion site is not conducive to guiding regenerating axons in the rostral‐caudal direction to facilitate synaptic reconnection. Hence, directing axonal growth and activating the regenerative potential of transplanted cells within the surrounding inhibitory glial scars remains a major unresolved issue, significantly limiting the therapeutic potential for treating SCI.

**Scheme 1:**
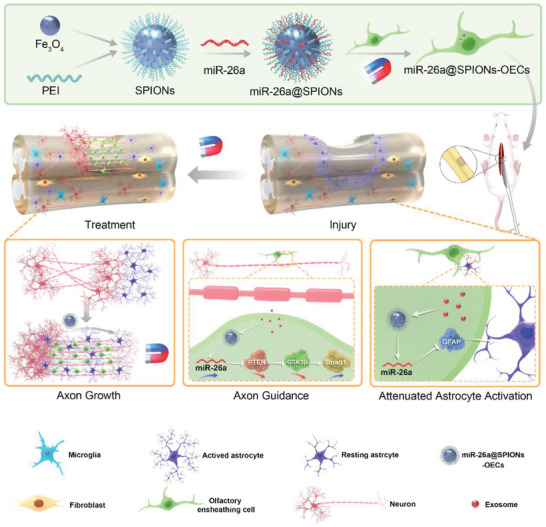
Schematic representation of miR‐26a@SPIONs‐OECs for spinal cord injury repair. Modified OECs mixed with Hyaluronic acid Hydrogel are injected in situ at the site of injury to promote spinal cord injury repair by directing axonal directional alignment to break through astrocyte scarring under the action of a magnetic field.

Cell engineering offers a promising method for augmenting cells with additional biological functionalities to surpass their intrinsic limitations in supporting tissue regeneration.^[^ ^13^ ^]^ In this study, we engineered OECs with miR‐26a‐loaded functionalized superparamagnetic iron oxide nanoparticles (SPIONs). The resulting miR‐26a@SPIONs‐OECs show a high sensitivity to magnetic fields (MF), offering synergistic multimodal cues to enhance neuronal regeneration under MF. First, under MF (ON), miR‐26a@SPIONs‐OECs deliver exogenous miR‐26a, which binds to SPIONs and enters dorsal root ganglion (DRG) or astrocytes via extracellular vesicles (EVs) from OECs. This delivery inhibits PTEN and GSK‐3β signaling pathways in neurons,^[^ ^14^ ^]^ thereby promoting neurite outgrowth. The application of MF augments the secretion of EV^miR‐26a@SPIONs‐OECs^ in vivo and in vitro. Second, under MF guidance magnetically responsive miR‐26a@SPIONs‐OECs migrate and orient along the MF direction. This orientation stimulates directional neurite elongation, aiding in neuronal reconnection and functional restoration. Third, miR‐26a enriched EVs from miR‐26a@SPIONs‐OECs downregulate the activation of surrounding astrocytes and, with the assistance of the applied MF, integrate with host astrocytes. This integration consequently minimizes inhibitory cues for cell survival and neurite growth in the surrounding environment. In sum, by combining structural guidance for axonal growth and the attenuation of surrounding glia, the miR‐26a@SPIONs‐OECs pro‐regeneration system introduces a potential methodology for central nervous system repair (**Scheme** **1**). It is worth noting that the strategy employed in this study is not limited to OECs for SCI but can be applied to other cells that are frequently used for numerous systemic disorders in clinics, including stem cells .

## 2 Results

### 2.1 Characterization of OECs Engineered with miR‐26a@SPIONs

**Figure 1:**
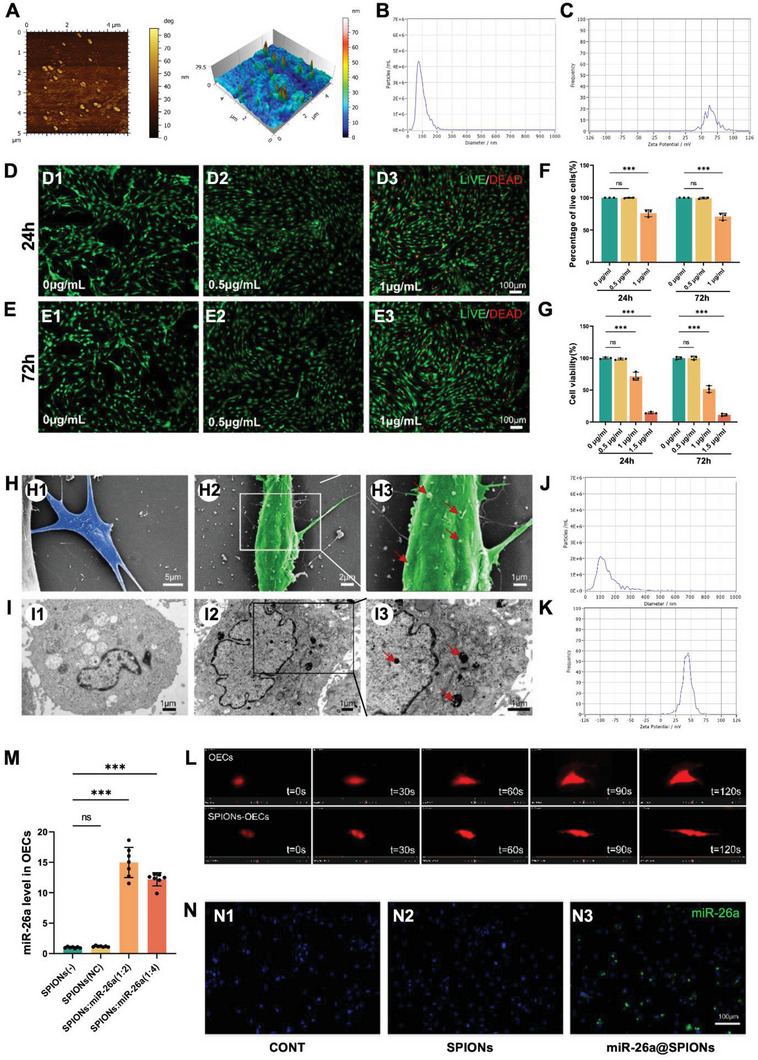
The characteristics of miR‐26a@SPIONs‐OECs. A) Atomistic microscopy images and 3D surface view of SPIONs. B) Representative nanoparticle analysis of the size distribution of SPIONs. C) Zeta potential of PEI‐SPIONs. Staining images of the LIVE/DEAD assay at 24 h D) and 72 h E) after transfection of OECs with different concentrations of SPIONs. F) The percentage of live cells was measured via the LIVE/DEAD assay. Live cells were stained green, while dead cells were stained red (*n* = 3, ***p < 0.001 for comparison with OECs without treatment). G) Cell viability was estimated using the CCK‐8 assay (*n* = 3, ***p < 0.001 for comparison with OECs without treatment). H)Representative SEM images of normal control OECs (H1) and OECs magnetofected with 0.5 µg mL^−1^ SPIONs for 24 h (H2). (H3) High magnification of the boxed area in (H2), The red arrows point to SPION agglomerates on the cell membrane. I) Representative TEM images of normal control OECs (I1) and OECs magnetofected with 0.5 µg mL^−1^ SPIONs for 24 h (I2). (I3) High magnification of the boxed area in (I2). The red arrows point to SPIONs in the cytoplasm; most retained SPIONs are dispersed as single particles. J) Overall distribution of miR‐26a@SPIONs complex sizes. K) Zeta potential of the miR‐26a@SPIONscomplex. L) Time‐series live imaging of OECs transfected with lentivirus^mCherry^ in CONT and SPIONs‐OECs group under the MF. M) At 72 h after magnetofection, miR‐26a levels in each group (normalized to normal control OECs) (*n* = 7, ***p < 0.001 for comparison with OECs without treatment). N) Representative in situ hybridization results of miR‐26a in OECs of different treatment groups. All statistical data were analyzed by one‐way ANOVA with Dunnett's multiple comparisons test and represented as mean ± SD, CONT = the control group, ns = no significance.

We evaluated the morphological features of PEI‐SPIONs using transmission electron microscopy (TEM) and noted an average diameter of 20.4 ± 2.6 nm (Figure S1A, Supporting Information). Subsequent analysis using atomic mechanical microscopy revealed smooth surfaces on the PEI‐SPIONs with no visible particles or pore structures. A 3D examination of their surface affirmed an average particle size of 79.5 ± 4.3 nm (**Figure** **1**A). Further measurements showed an average diameter of 84.8 ± 6.7 nm (Figure 1B) and a positive charge of 64.7 ± 3.6 mV (Figure 1C) for the PEI‐SPIONs. These findings indicate that PEI‐SPIONs acquire a positive charge upon binding to plasmids, making them suitable vehicles for gene transfer into cells.

The purity of OECs utilized in this study exceeded 95% (Figure S1B–E, Supporting Information). We determined the cytotoxicity of PEI‐SPIONs by performing LIVE/DEAD assays at 24 and 72 h post‐magnetofection (Figure 1D–F). At 24 h post‐magnetization, PEI‐SPIONs at a concentration of 0.5 µg mL^−1^ exhibited no toxic effects on OECs. However, upon increasing the concentration to 1 µg mL^−1^, cell mortality rose to 24.08 ± 5.38%. At 72 h post‐magnetization, PEI‐SPIONs at 0.5 µg mL^−1^ also exhibited no toxic effects on OECs, while at 1 µg mL^−1^, the mortality rate spiked to 29.28 ± 5.33%. The CCK‐8 assay provided further validation of the cytotoxicity of PEI‐SPIONs at concentrations ranging from 0 to 1.5 µg mL^−1^ (Figure 1G). Based on these findings, we selected 0.5 µg mL^−1^ as the optimal concentration for magnetofection of PEI‐SPIONs into OECs for this study.

Our subsequent investigation was to discern whether PEI‐SPIONs could be internalized by OECs. We observed PEI‐SPIONs (0.5 µg mL^−1^) on the OEC surface after 6 h of magnetofection (Figure 1H). Post 24 h of magnetization, TEM analysis revealed clusters of nanoparticles within the OECs (Figure 1I), which suggested that PEI‐SPIONs could adhere to the cells and penetrate the cytoplasm. Once the PEI‐SPIONs were functionalized with miR‐26a, the average diameter increased to 102.7 ± 1.4 nm, while the positive charge dropped to 44.1 ± 2.5 mV (Figure 1J,K). These results implied that SPIONs‐(miR‐26a) can bind to positively charged plasmids, are suitable for transfection into OECs, and may enhance miR‐26a expression within OECs. Furthermore, OECs were labeled with the mCherry, and the live imaging showed that the migration process of SPIONs‐OECs tended to be more oriented under the MF (Figure 1L, Videos S1 and S2, Supporting Information).

We observed a significant augmentation of miR‐26a levels in OECs treated with miR‐26a@SPIONs, particularly when the weight ratio of PEI‐SPIONs to plasmid was set at 1:2 (Figure 1M). In addition, the miR26a level was not altered in OECs‐EVs loaded with SPIONs vector, indicating that SPIONs alone had a negligible effect on EVs physiology. Further, in situ hybridization confirmed the presence of miR‐26a exclusively in OECs within the miR‐26a@SPIONs group (Figure 1N), thus validating the successful transfer of miR‐26a to OECs via the miR‐26a@SPIONs system.

### 2.2 Enhanced Axonal Growth in DRG Neurons by miR‐26a@SPIONs‐OECs through EVs Traffic

**Figure 2:**
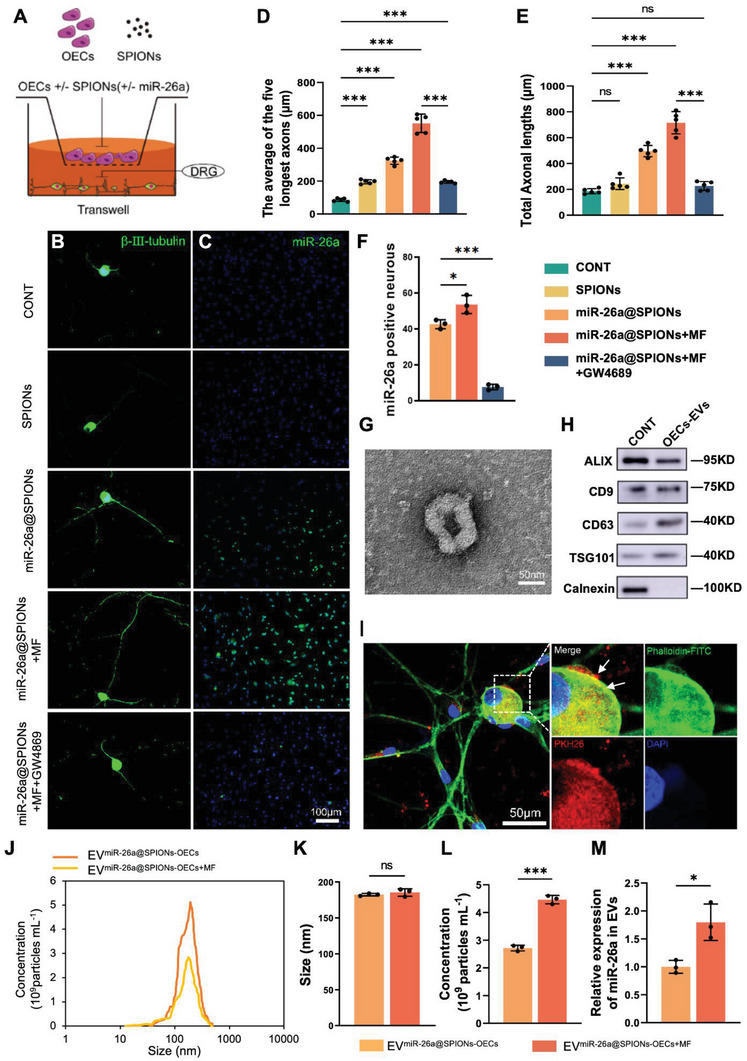
miR‐26a@SPIONs‐OECs enhance the axonal growth of DRG neurons. A) Schematic diagram of OECs co‐cultured with DRG neurons. B) Axonal elongation of DRGs which co‐cultured with OECs in different treatment groups after 2 days. Representative images of DRG neurons were stained for β‐III‐tubulin (green). C) In situ hybridization of miR‐26a in DRG with co‐cultures of OECs from different treatment groups. D,E) Axonal lengths were quantified and plotted for each treatment of five independent experiments (*n* = 5, ***p < 0.001 for comparison with OECs without treatment). F) Quantifications of the total number of miR‐26a‐positive DRG neurons in different treatment groups (*n* = 3, *p < 0.05 and ***p < 0.001 for comparison with OECs without treatment). G) TEM analysis of isolated EVs. H)Western blot analysis of miR‐26a@SPIONs‐OECs‐EVs confirmed the presence of EV marker proteins of ALIX, CD9, TSG101, and the absence of non‐exosomal protein (calnexin), proteins were quantified by BCA protein assay and 2 µg of OECs lysate or 2 µg of OECs‐EVs were loaded in each lane. I) The miR‐26a@SPIONs‐OECs‐EVs were labeled with PKH26 (red), and DRG neurons were immunofluorescent stained, for Phalloidin (green) with DAPI nuclear counterstaining (blue). J) The overall distribution of EV^miR‐26a@SPIONs‐OECs^ and EV^miR‐26a@SPIONs‐OECs+MF^. Size K) and quantification L) analyses of EV^miR‐26a@SPIONs‐OECs^ and EV^miR‐26a@SPIONs‐OECs+MF^ by NTA (*n* = 3, ***p < 0.001 for comparison with EV^miR‐26a@SPIONs‐OEC^). M) Detection of miR‐26a content in EVs by qPCR (*n* = 3, *p < 0.05 for comparison with EV^miR‐26a@SPIONs‐OEC^). D–F) were analyzed by one‐way ANOVA with Dunnett's multiple comparisons test and (K‐M) were analyzed by Student's *t*‐test. All statistical data were represented as mean ± SD, CONT = the control group, ns = no significance.

The DRG neuron is an invaluable tool in axon growth and myelin formation research. We established an optimized immunopanning method to purify DRG neurons and reached a purity of 94%. In this portion of our study, we investigated the influence of miR‐26a@SPIONs‐OECs on the neurite outgrowth of purified DRG neurons (**Figure** **2**A). After co‐culturing the neurons with miR‐26a@SPIONs‐OECs in Transwell Petri dishes for 24 h, we observed a modest increase in the neurite growth of DRG neurons, even without applying an MF (Figure 2B). However, when we applied an MF, there was a further enhancement of the neurite length of the co‐cultured neurons (Figure 2D,E).

We also performed an in situ hybridization assay, which revealed a significant increase in miR‐26a expression in DRG neurons co‐cultured with miR‐26a@SPIONs‐OECs. This expression was further amplified when an MF was applied (Figure 2C,F).

To understand how miR‐26a@SPIONs‐OECs+MF boosted neurite growth and miR‐26a levels in DRG neurons, we proposed a paracrine mechanism for cellular communication between OECs and DRGs, as these were cultured in separate compartments. Consequently, we collected EVs following standard protocols from the supernatant in a typical paracrine mode. The vesicles displayed a modal peak of 182.4 ± 1.7 nm (Figure 2J,K) and a cup‐shaped morphology characteristic of EVs (Figure 2G). Additionally, the EVs tested positive for the exosomal markers ALIX, CD63, and TSG101, but not for calnexin (Figure 2H). To further explore the role of EVs in this process, we apply an EV synthesis inhibitor GW4869 to the culture system. Both the miR‐26a in neurons and neurite outgrowth were significantly compromised in the miR‐26a@SPIONs‐OECs group, which further confirmed that the pro‐regenerative effects were mainly due to EVs. (Figure 2B–F). Notably, the miR‐26a in EVs derived from miR‐26a@SPIONs‐OECs was nearly 1.7‐fold higher than that derived from SPIONs‐OECs. Additionally, in the presence of MF, the mechanical cues significantly boosted the EV production and miR‐26a level in a whole (Figure 2L,M).

We then traced the EVs' location using the PKH26 label to exhibit the relationship between EVs and DRG neurons. After 8 h of incubation, we detected PKH26‐labeled EVs in the cell bodies of DRG neurons (Figure 2I). This finding suggests that EVs can be internalized by DRG neurons and that miR‐26a can be transported from miR‐26a@SPIONs‐OECs to DRG neurons via EVs. This process is further intensified by the application of MF.

### 2.3 Axonal Outgrowth Promotion by EVs Derived from miR‐26a@SPIONs‐OECs via PTEN/GSK‐3β/SMAD1 Axis Activation in Neurons

We first utilized MiRWalk and Metascape to functionally annotate and enrich the target genes of miR‐26a, to predict the cell‐specificity of genes enriched for miR‐26a. 837 genes that could bind to miR‐26a were identified via the miRWalk database (Figure S2A, Supporting Information). The results showed that miR‐26a can target multiple genes, which were involved in multiple biological processes (Figure S2B, Supporting Information). To further investigate the expression patterns of these genes across different cells and tissues, we then consulted the PaGenBase database to predict the target genes of mir‐26a that might function predominantly in DRG neurons (Enrichment score = 3.3/Z‐score = 3.9). Further analyses showed that miR‐26a is conserved across species and exhibits gene regulation functions on DRG neurons. Moreover, our findings revealed miR‐26a's involvement in the normal life activities of the organism, such as the biological regulation of growth and development. Additionally, we found that miR‐26a primarily plays a role in diseases related to central nervous system injury (Figure S2C,D, Supporting Information).

**Figure 3:**
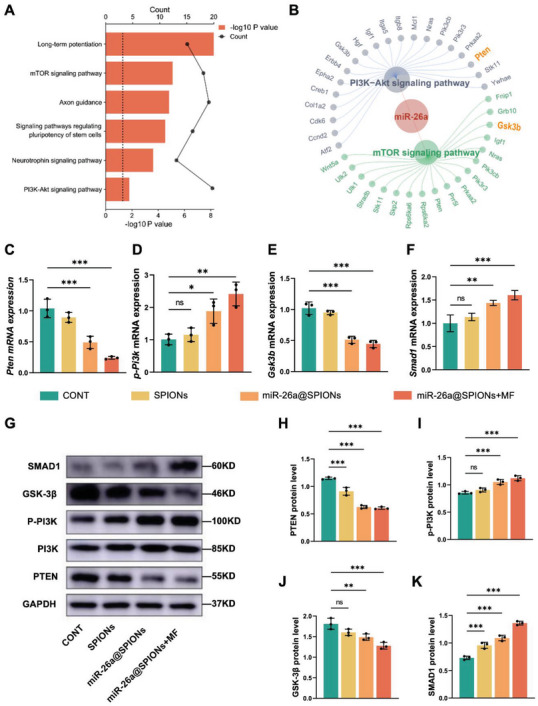
Characteristics of miR‐26a@SPIONs‐OECs‐EVs and their internalization by DRG neurons. A) GO enrichment analysis of miR‐26a. B) KEGG enrichment analysis of miR‐26a. C) *Pten*, D) *p‐Pi3k*, E) *Gsk3b*, and F) *Smad1* mRNA expression in different treatment DRGs after 2 days (*n* = 3, *p < 0.05, **p < 0.01 and ***p < 0.001 for comparison with OECs without treatment). G) Detection of GAPDH, PTEN, P‐PI3K, GSK‐3β, SMAD1 protein level in CONT, SPIONs, miR‐26a@SPIONs and miR‐26a@SPIONs+MF by Western blotting. H) PTEN, I) P‐PI3K, J) GSK‐3β, and K) SMAD1 protein level in different treatment DRGs after 2 days of (G) (*n* = 3, **p < 0.01 and ***p < 0.001 for comparison with OECs without treatment). All statistical data were analyzed by one‐way ANOVA with Dunnett's multiple comparisons test and represented as mean ± SD, CONT = the control group, ns = no significance.

Through KEGG and GO analyses, we identified the main pathways highly enriched by miR‐26a, including the mTOR, axon guidance, and PI3K‐AKT signaling pathways (**Figure** **3**A,B). Our study concentrated on the PTEN/GSK‐3β/Smad1 pathway, known to be critical for axonal regeneration.^[^ ^15^ ^]^ Interestingly, we observed a down‐regulation of *Pten* and *Gsk3b* mRNA levels, while *p‐Pi3k* and *Smad1* mRNA levels were up‐regulated in DRG neurons co‐cultured with miR‐26a@SPIONs‐OECs (Figure 3C–F). Western blotting further confirmed the protein expression levels of PTEN, p‐PI3K, GSK‐3β, and SMAD1 (Figure 3G–K). These results suggest that miR‐26a derived from miR‐26a@SPIONs‐OECs inhibits PTEN and GSK‐3β and their downstream.

We further evaluated the role of *Pten* by overexpressing it in DRG neurons (*Pten^+/+^*) and co‐culturing these with miR‐26a@SPIONs‐OECs (Figure S3A, Supporting Information). After 24 h, we observed that *Pten^+/+^* nullified the effect of miR‐26a and significantly decreased neurite length in DRG neurons (Figure S3B–G, Supporting Information). *Pten^+/+^* also significantly upregulated *Gsk3b* while downregulating *p‐Pi3k* and *Smad1* at both the mRNA (Figure S3H–K, Supporting Information) and protein levels in neurons (Figure S3L–P, Supporting Information). These results suggest that miR‐26a derived from OECs promotes neurite growth via the PTEN/GSK‐3β/Smad1 pathway.

### 2.4 Alignment of miR‐26a@SPIONs‐OECs Promotes Axonal Growth and Directs Neurite Elongation

Once the beneficial impacts of miR‐26a on neurite growth were affirmed, we delved into the ability of the MF to align and migrate miR‐26a@SPIONs‐OECs in vitro. An Orientation Index (Oi), introduced for the analysis, served to evaluate OECs' alignment (Figure S4A, Supporting Information). The Oi represents the cosine value of the long axis of OECs and the external MF direction's angle θ. If 0 < θ < 1, Oi = 1, indicating the migration direction of OECs aligns with the external MF direction. In this study, the miR‐26a@SPIONs‐OECs showed an orientation along the MF direction, with an Oi of 0.97 ± 0.05, in marked contrast to the other groups (Figure S4B–H, Supporting Information). Additionally, the MF led to an increase in the migration distance of SPIONs‐OECs, a significant improvement compared to SPIONs‐OECs without MF and OECs with MF (Figure S4I, Supporting Information). This evidence suggests that the MF can enhance the in vitro migration and alignment of miR‐26a@SPIONs‐OECs.

**Figure 4:**
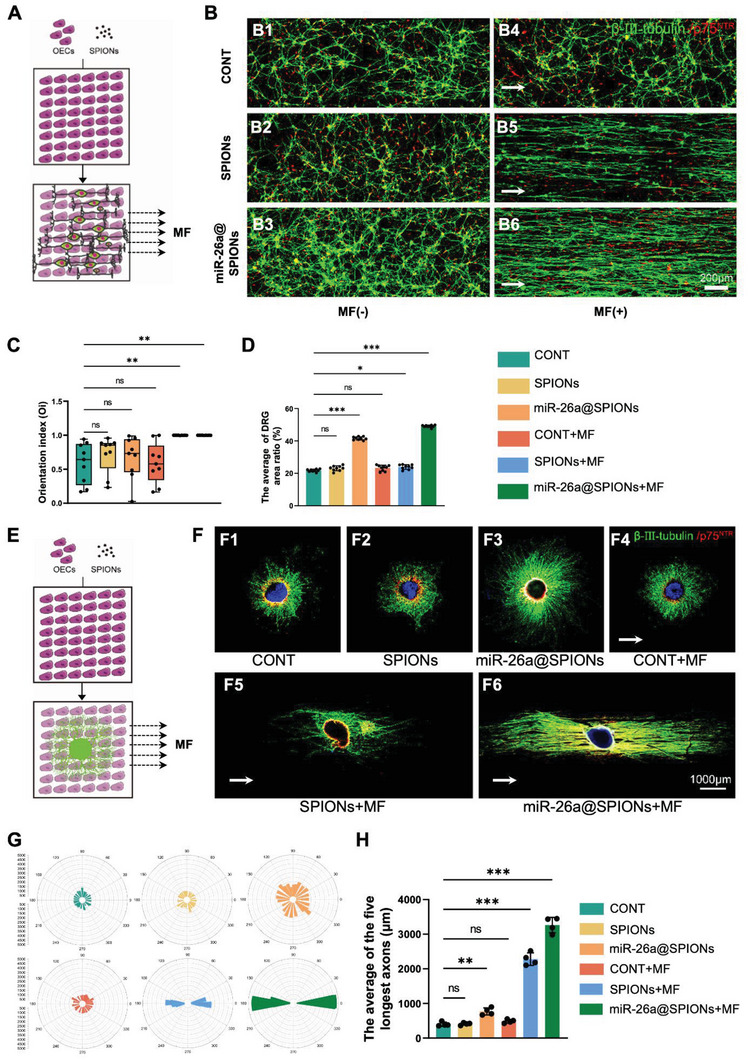
Directional growth of DRG in co‐culture with OECs. A)Schematic diagram of the growth of DRG neurons in neurons on the OECs layer; B) Immunofluorescence staining of the directed growth of DRG neurons in different treatment groups; OECs specifically expresses p75^NTR^ (red), and DRG neuron specifically expresses β‐III‐tubulin (green); C) DRGs migration orientation index. The box plot showed the median, interquartile ranges, maximum, and minimum (*n* = 9, **p < 0.01 for comparison with OECs without treatment). D) The average DRG area ratio (*n* = 9, *p < 0.05 and ***p < 0.001 for comparison with OECs without treatment). E) DRG explants on the OECs layer growth diagram; F)Immunofluorescence staining of DRG explants growth in different treatment groups, specific expression of p75^NTR^ by OECs (red), β‐III‐tubulin specific expression by DRG neurons (green), and the nucleus is stained with DAPI (blue); G) Statistical maps of DRG explants orientation in different treatment groups; H) Statistical diagram of the average length of the longest 5 axons of DRG explants in different treatment groups (*n* = 4, **p < 0.01 and ***p < 0.001 for comparison with OECs without treatment). All statistical data were analyzed by one‐way ANOVA with Dunnett's multiple comparisons test and represented as mean ± SD, CONT = the control group, ns = no significance.

We further investigated how the alignment of miR‐26a@SPIONs‐OECs impacted the orientation of neurite growth in purified DRG neurons (**Figure** **4**A). In OECs without significant alignment (including OECs with SPIONs or miR‐26a@SPIONs and OECs with an MF), neurites of neurons displayed a network‐like pattern. However, in well‐aligned OECs (miR‐26a@SPIONs+MF), the neurites followed the direction of OECs alignment (Figure 4B,C). This evidence hints that OEC alignment could guide the orientation of neurites in neurons. Moreover, the DRG neurites' density was significantly higher in miR‐26a@SPIONs‐OECs, whether or not an MF was present (Figure 4B,D), further emphasizing miR‐26a's positive influence on neurite growth.

Finally, we validated the beneficial effects of miR‐26a@SPIONs‐OECs on neurite alignment and growth in DRG explants (Figure 4E). Similarly, to the purified neuron system, the neurites followed the same direction as the OECs alignment in the miR‐26a@SPION‐OECs+MF group. This result contrasts with the random pattern of neurite growth in OECs without significant alignment (Figure 4F,G). Furthermore, treatment of OECs with either miR‐26a or MF led to a significant increase in neurite length (Figure 4F,H), reinforcing the positive influence of miR‐26a@SPIONs‐OECs on neurite growth and alignment in neurons.

### 2.5 miR‐26a@SPIONs‐OECs Promote Directed Migration on Astrocyte Monolayers

**Figure 5:**
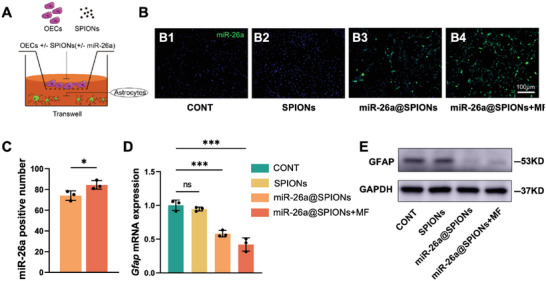
Effect of different treatment groups of OECs on astrocytes. A) Schematic diagram of astrocytes co‐cultured with OECs. B) Graph of the results of in situ hybridization experiments with different groups of astrocytes. C) Statistics of miR‐26a‐positive cells in astrocytes of different treatment groups of Figure B (*n* = 3, *p < 0.05 for comparison with miR‐26a@SPIONs‐OECs. Data were analyzed by Student's *t*‐test). D) *Gfap* mRNA expression in different treatment astrocytes after 2 days (*n* = 3, ***p < 0.001 for comparison with OECs without treatment. Data were analyzed by one‐way ANOVA with Dunnett's multiple comparisons test). (E) Detection of GFAP protein level in CONT, NC@SPIONs, miR‐26a@SPIONs, and miR‐26a@SPIONs+MF by Western blotting. All statistical data were represented as mean ± SD, CONT = the control group, ns = no significance.

Following SCI, astrocytes become activated, proliferating to form glial scars that surround implanted cells and create a physical barrier impeding neural cell growth, resulting in axon regeneration failure. Hence, we explored the impact of miR‐26a@SPIONs‐OECs on astrocytes (**Figure** **5**A). We first isolated astrocytes from neonatal mice (Figure S5A–C, Supporting Information) and co‐cultured them with miR‐26a@SPIONs‐OECs in Transwell Petri dishes. In situ hybridization revealed an abundance of miR‐26a expression in astrocytes co‐cultured with miR‐26a@SPIONs‐OECs (Figure 5B). Notably, MF application further augmented miR‐26a levels in astrocytes (Figure 5C). Concurrent studies showed that *Gfap* mRNA and protein levels decreased in correlation with the rise in miR‐26a levels in astrocytes (Figure 5D,E). As GFAP protein is a specific marker for activated astrocytes, it is plausible that OEC‐derived EVs transfer miR‐26a to astrocytes, inhibiting astrocyte activation and thereby reducing scar production. This action potentially creates a regeneration‐friendly environment for injured axons.

**Figure 6:**
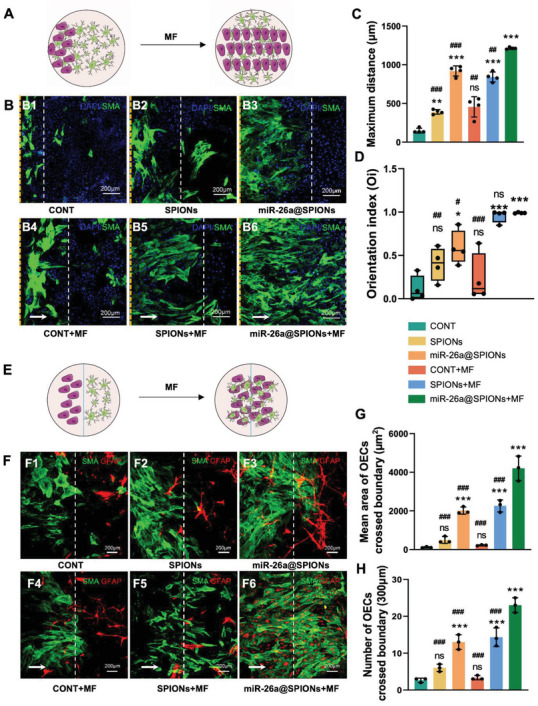
Effect of different treatment groups of OECs on migration and orientation of astrocytes. A) Schematic diagram of OEC migration on an astrocyte monolayer in the inverted coverslip migration assay. B) Immunocytochemical image of OEC growth on the astrocyte layer. OECs were labeled with SMA (green) and DAPI (blue), while astrocytes were labeled with the nucleus staining solution DAPI (blue) only. White arrows indicate the direction of the applied MF. The yellow line is the starting point of cell migration, and the white line is the end point of cell migration. C) Average maximum distances of migration from the edge of the inverted fragments (*n* = 4, **p < 0.01 and ***p < 0.001 for comparison with OECs without treatment; ##*p* < 0.01 and ###*p* < 0.001 for comparison with miR‐26a@SPIONs‐OECs+MF). D) OEC migration orientation index. The box plot showed the median, interquartile ranges, maximum, and minimum (*n* = 4, *p < 0.05 and ***p < 0.001 for comparison with OECs without treatment, #*p* < 0.05, ##*p* < 0.01, and ###*p* < 0.001 for comparison with miR‐26a@SPIONs‐OECs+MF). E) Schematic diagram of the OEC–astrocyte confrontation assay. Magnetofected OECs and astrocytes were plated in parallel strips; both were allowed to grow and migrate for 48 h under an applied MF. F) Immunocytochemistry images of the OEC–astrocyte confrontation assay. OECs were labeled with SMA (green); while astrocytes were labeled with GFAP (red). Arrows indicated the direction of the MF. The white line is midway between the two types of cells. G) The mean area of OECs that crossed the boundary and migrated into the astrocyte domain (*n* = 3, ***p < 0.001 for comparison with OECs without treatment, ###*p* < 0.001 for comparison with miR‐26a@SPIONs‐OECs+MF). H) Quantification of OECs that crossed the boundary and migrated into the astrocyte domain (*n* = 3, ***p < 0.001 for comparison with OECs without treatment, ###*p* < 0.001 for comparison with miR‐26a@SPIONs‐OECs+MF). All statistical data were analyzed by one‐way ANOVA with Dunnett's multiple comparisons test and represented as mean ± SD, CONT = the control group, ns = no significance.

Next, we examined how MF influenced the migration and orientation of miR‐26a@SPIONs‐OECs on an astrocyte monolayer (**Figure** **6**A). In the CONT group, CONT+MF group and SPIONs@OECs group, migration was unaffected on astrocytes. After magnetofection with miR‐26a, a significant increase was seen in the migration distance of miR‐26a@SPIONs‐OECs, but without a directed orientation in the absence of an MF (Figure 6B–D). However, in the presence of an MF, the migration distance of miR‐26a@SPIONs‐OECs further increased on the astrocyte monolayer. Interestingly, the miR‐26a@SPIONs‐OECs followed the direction of the MF, a sharp contrast to the random distribution seen in OECs without an MF. Collectively, these findings suggest that an MF enhances the migration and orientation of miR‐26a@SPIONs‐OECs on an astrocyte monolayer, potentially guiding neurite growth following SCI.

We executed a confrontation assay to determine whether miR‐26a@SPIONs‐OECs could migrate across the astrocyte boundary and infiltrate the astrocyte domain under MF application (Figure 6E). Visible cell boundaries existed between the astrocytes and OECs in most groups. However, with the application of an MF, miR‐26a@SPIONs‐OECs successfully crossed the astrocyte‐formed boundary. Guided by the MF, the miR‐26a@SPIONs‐OECs migrated in the direction of the MF axis and amalgamated with astrocytes. The highest area and number of OECs crossing the boundary and migrating into the astrocyte regions were seen in the miR‐26a@SPIONs‐OECs+MF group, followed by the SPIONs‐OECs+MF and miR‐26a@SPIONs‐OECs groups; the least were seen in the remaining groups (Figure 6F–H). These results illustrate that OECs functionalized by SPIONs and miR‐26a can interact with central glial cells, demonstrating an enhanced ability to integrate with astrocytes under the influence of an MF.

### 2.6 miR‐26a@SPIONs‐OECs Improved Nerve Regeneration and Functional Restoration after SCI In Vivo

**Figure 7:**
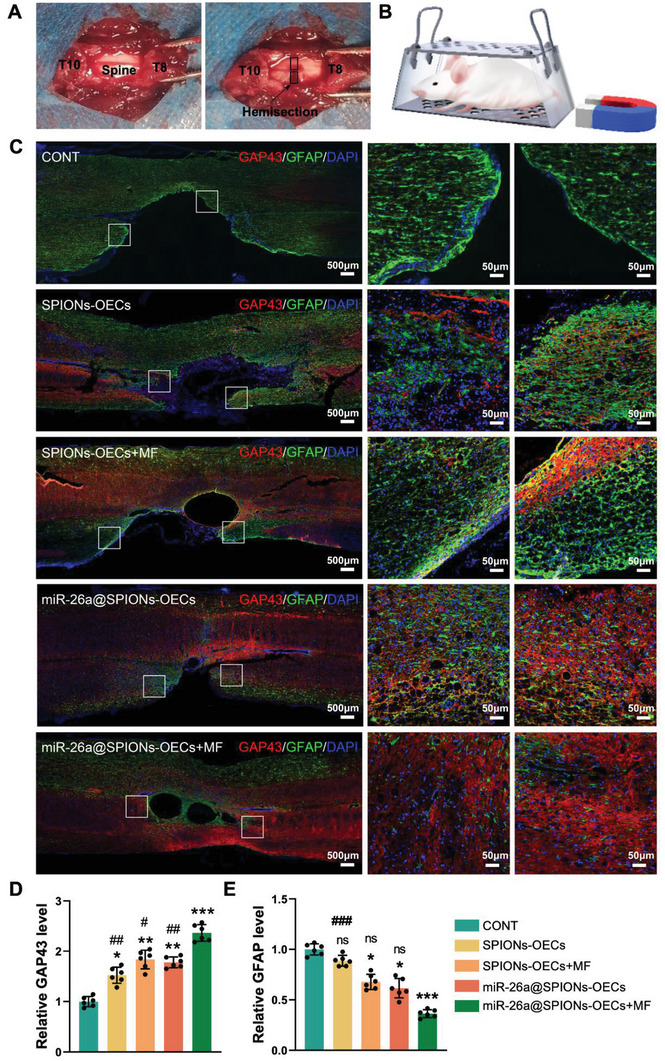
Establishment and treatment of hemisection modeling of spinal cord injury. A) The T9 spinous process and lamina were dissected with a bone biter to expose the spinal cord. Use microsurgical scissors to the transaction from the right half of the spinal cord to the median venous margin. B) Schematic illustration of the therapeutic experiment. C) Immunofluorescence double staining of GAP43 (red) and GFAP (green) proteins in different groups at week 8. D,E) Statistical analysis of the fluorescence intensity of GAP43 and GFAP (*n* = 6, *p < 0.05, **p < 0.01 and ***p < 0.001 for comparison with OECs without treatment; #*p* < 0.05, ##*p* < 0.01 and ###*p* < 0.001 for comparison with miR‐26a@SPIONs‐OECs+MF). All statistical data were analyzed by one‐way ANOVA with Dunnett's multiple comparisons test and represented as mean ± SD, CONT = the control group, ns = no significance.

We assessed the therapeutic potential of miR‐26a@SPIONs‐OECs in a rat model of spinal cord hemisection with a lesion gap of 2 ± 0.2 mm (**Figure** **7**A). In the lesion site, we implanted 5 × 10^5^ miR‐26a@SPIONs‐OECs using Hyaluronic acid Hydrogel and then exposed them to an MF (1.4T, 2 h day^−1^ for 8 weeks) (Figure 7B). We divided the animals into six groups: 1) The sham group, operated mice without SCI; 2) The CONT group, untreated SCI rats; 3) The SPIONs‐OECs group, the SCI rats treated with SPIONs‐OECs; 4) The SPIONs‐OECs+MF group, the SCI rats treated with both SPIONs‐OECs and MF; 5) The miR‐26a@SPIONs‐OECs group, the SCI rats treated with miR‐26a@SPIONs‐OECs; and 6) The miR‐26a@SPIONs‐OECs+MF group, the SCI rats treated with both miR‐26a@SPIONs‐OECs and MF.

To gain a better understanding of the structural restoration across each group, we stained sagittal sections of the injured spinal cord with GFAP (to visualize activated astrocytes) and neuromodulin GAP43 proteins (indicating newly regenerated nerve fibers) (Figure 7C). The CONT group displayed a high density of GFAP‐positive astrocytes at the lesion edge 56 days post‐surgery, signifying a glial scar around the lesion site. In contrast, miR‐26a@SPIONs‐OECs effectively limited the overactivity and accumulation of astrocytes during secondary injury and instigated an extensive redistribution of long nerve fibers. The density ratio of GAP43 to GFAP at the lesion edge was reversed by miR‐26a@SPIONs‐OECs (Figure 7D,E). The use of MF treatment further augmented the beneficial effects of miR‐26a@SPIONs‐OECs in SCI, demonstrated by a reduced area of focal damage and a higher GAP43 to GFAP fluorescence ratio. These findings suggest that by promoting the regeneration of new nerve bundles and inhibiting glial scar formation, miR‐26a@SPIONs‐OECs enhances histological restoration after SCI.

**Figure 8:**
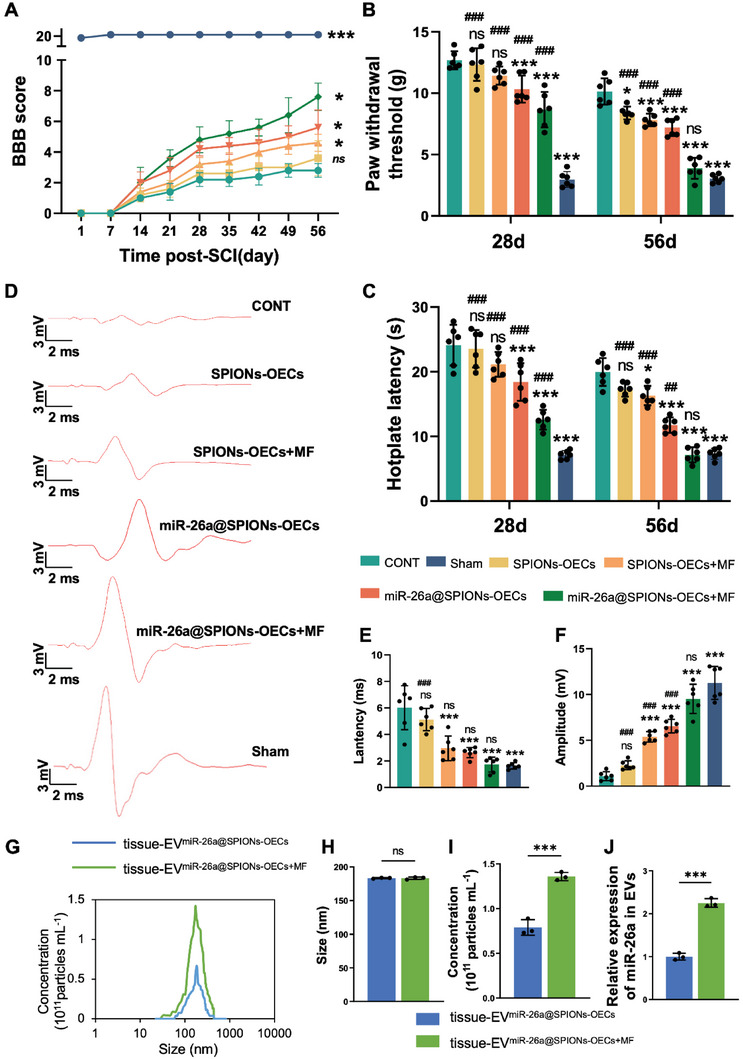
Motor functional recovery of SCI rats after treatment with the miR‐26a@SPIONs‐OECs under MF. A) Basso, Beattie & Bresnahan locomotor rating scale of hindlimbs of mice from different groups during the 8‐week treatment periods (*n* = 5, *p < 0.05 and ***p < 0.001 for comparison with OECs without treatment). B) The paw withdrawal threshold of CONT, Sham, SPIONs‐OECs, SPIONs‐OECs+MF, miR‐26a@SPIONs and miR‐26a@SPIONs+MF (*n* = 6, *p < 0.05 and ***p < 0.001 for comparison with OECs without treatment; ###*p* < 0.001 for comparison with miR‐26a@SPIONs‐OECs+MF). C) The Hotplate latency of CONT, Sham, SPIONs‐OECs, SPIONs‐OECs+MF, miR‐26a@SPIONs and miR‐26a@SPIONs+MF (*n* = 6, *P < 0.05 and ***P < 0.001 for comparison with OECs without treatment; ##*p* < 0.01 and ###*p* < 0.001 for comparison with miR‐26a@SPIONs‐OECs+MF). D) Representative images of MEPs 28 days and 56 days post‐surgery. E)The latency of MEPs (*n* = 6, ***p < 0.001 for comparison with OECs without treatment; ###*p* < 0.001 for comparison with miR‐26a@SPIONs‐OECs+MF). F)The peak amplitude of MEPs (*n* = 6, ***p < 0.001 for comparison with OECs without treatment; ###*p* < 0.001 for comparison with miR‐26a@SPIONs‐OECs+MF). G)The overall distribution of tissue‐EV^miR‐26a@SPIONs‐OECs^ and tissue‐EV^miR‐26a@SPIONs‐OECs+MF^ from the spinal cord. Size H) and quantification I) analyses of tissue‐EV^miR‐26a@SPIONs‐OECs^ and tissue‐EV^miR‐26a@SPIONs‐OECs+MF^ by NTA (*n* = 3, ***p < 0.001 for comparison with EV^miR‐26a@SPIONs‐OECs^). J) Detection of miR‐26a content in EVs by qPCR (*n* = 3, ***p < 0.001 for comparison with EV^miR‐26a@SPIONs‐OECs^). A–C) and (E,F) were analyzed by one‐way ANOVA with Dunnett's multiple comparisons test and H–J) were analyzed by Student's *t*‐test. All statistical data were represented as mean ± SD, CONT = the control group, ns = no significance.

We performed behavioral analysis to evaluate motor function recovery post‐SCI in each group. The CONT group rats exhibited near‐complete paralysis, with a Basso‐Beattie‐Bresnahan (BBB) score of 1.75 ± 0.66 on day 56 post‐surgery (**Figure** **8**A). Compared with the sham group (20.73 ± 0.8), we observed a partial motor function improvement in the SPIONs‐OECs (1.96 ± 1.33), SPIONs‐OECs+MF (2.56 ± 1.79), and miR‐26a@SPIONs‐OECs (3.18 ± 2.11) groups. However, the miR‐26a@SPIONs‐OECs +MF group achieved a significantly higher BBB score of 3.91 ± 2.72, suggesting that miR‐26a@SPIONs‐OECs under MF enhances motor function recovery in the SCI model. Motor‐evoked potentials electrical stimulation further confirmed the beneficial impact of the pro‐regeneration system on motor functional recovery post‐SCI (Figure 8D). The miR‐26a@SPIONs‐OECs+MF group animals displayed the shortest response latency (1.73 ± 0.56 ms) and the highest amplitude (9.52 ± 1.59 mV) closest to the sham group, as compared to the remaining groups (Figure 8E,F).

Sensory perturbations are frequent and debilitating complications of SCI. Therefore, we evaluated changes in evoked pain using the von Frey filament test (Figure 8B) and hot plate test (Figure 8C). At 28 days post‐surgery, we observed no substantial difference in mechanical sensitivity between the CONT, SPIONs‐OECs, and SPIONs‐OECs+MF groups (*P* > 0.05). However, the miR‐26a@SPIONs‐OECs+MF group (8.66 ± 1.45 g) had a significantly lower paw withdrawal threshold (*P* < 0.001), even compared with the sham group (2.97 ± 0.64 g), the disparity remains. At 56 days post‐operation, the paw withdrawal threshold significantly decreased in all treatment groups compared to the CONT group, with the lowest in the miR‐26a@SPIONs‐OECs+MF group (3.88 ± 0.86 g). For the hot plate test, all groups showed a significant decrease in paw retraction latency compared to the CONT group (19.97 ± 2.15 s) at 28‐ and 56 days post‐operation. Notably, the shortest reaction latency to the hot plate was seen in the miR‐26a@SPIONs‐OECs+MF group (7.15 ± 1.18 s). These results demonstrate that miR‐26a@SPIONs‐OECs effectively restore sensory disorders post‐SCI by increasing sensitivity to both mechanical and thermal stimuli.

To bear out the OEC's response to the magnetic stimulation in vivo, we further collected EVs from the local injury site. It was found that production of EVs collected from the miR‐26a@SPIONs‐OECs+MF group was 1.72‐fold higher than that of the miR‐26a@SPIONs‐OECs group (Figure 8G–I), indicating that mechanical force plays a vital role in promoting EVs secretion in vivo. In addition, we found that miR‐26a levels in EVs from the miR‐26a@SPIONs‐OECs+MF group were 2.25‐fold higher than the miR‐26a@SPIONs‐OECs group (Figure 8J), which further confirmed that the in vivo miR‐26a@SPIONs‐OECs could react to MF by increasing EVs production.

## 3 Discussion

SCI is a debilitating condition that lacks effective treatment options due to the limited regenerative capacity of adult CNS neurons and the hostile injury environment.^[^ ^16^ ^]^ Consequently, axonal growth and functional recovery after SCI are significantly compromised.^[^ ^17^ ^]^ Although numerous strategies have targeted extrinsic axonal regeneration mechanisms such as extracellular inhibitory molecule removal, neurotrophic factor delivery, and permissive substrate grafting, substantial functional recovery and robust regeneration of injured axons have remained elusive.^[^ ^18^ ^]^ This study introduces a pro‐regenerative system designed to provide external bioactive cues to encourage linear axonal growth, moderate local astrocyte activation, and ultimately improve both morphological and functional recovery following SCI.

OECs are specialized glial cells located in the olfactory system, that guide axonal growth from the periphery to the central nervous system.^[^ ^19^ ^]^ The ongoing adult neurogenesis in the olfactory bulb underscores the potential of OECs for CNS repair.^[^ ^20^ ^]^ However, OECs and SCs belong to the same family of glial cells, and they express many same phenotypic markers, like p75^NTR^, GFAP, and S100.^[^ ^21^ ^]^ Even though, there are still scientific methods to differentiate them. Previous studies have provided direct in vitro and in vivo evidence demonstrating that p75^NTR^‐positive OECs express SMA, whereas p75^NTR^‐positive SCs do not.^[^ ^22^ ^]^ This makes it possible to differentiate SCs and OECs through their discrepancy in expressing biomarkers. Evidence suggests that OECs can enhance neurite outgrowth, promote remyelination, provide guidance cues, and modulate local immunity.^[^ ^23^ ^]^ Moreover, OECs can be easily harvested from the nasal mucosa, enabling potential autologous transplantation. A recent clinical trial reported sensorimotor improvements after autologous mucosal OEC transplantation in patients with AIS grade A injury.^[^ ^23^ ^]^ However, a significant issue is the poor integration of transplanted OECs into the astrocyte‐rich CNS due to their limited motility within the CNS environment.^[^ ^24^ ^]^ This inadequate integration impedes axonal guidance by the transplanted OECs and, paradoxically, may trigger increased astrocyte activation at the injury site.^[^ ^25^ ^]^

In the context of successful CNS regeneration, transplanted OECs must cross the OEC‐astrocyte boundary and form an aligned structure that guides regenerating axons to their target. Over the past few decades, several strategies, such as the use of aligned‐structure biomaterials,^[^ ^4a^ ^]^ chemotactic induction,^[^ ^26^ ^]^ and physical cues^[^ ^27^ ^]^ have been proposed to enhance local cell orientation. Despite these efforts, orienting cell migration and alignment in the CNS remains a significant challenge due to the formation of OEC‐astrocyte borders. This study employed SPIONs as mechanical cues, guiding OEC migration and alignment under an MF. With proper concentration, SPIONs can efficiently endow cells with targeting abilities stemming from their remarkable properties (magnetic property^[^ ^28^ ^]^ and low toxicity) and selectively deliver cells toward targeted locations under an MF.^[^ ^29^ ^]^ Recent research indicated that SPIONs were incorporated by neurons and a tensile force stimulated neurite initiation and axon elongation in the desired direction,^[^ ^30^ ^]^ indicating that SPIONs are capable of responding to the magnetic field, and their actions have been widely utilized due to their high tissue‐penetrative, biocompatible, and spatiotemporally controllable characteristics. In the present study, the SPIONs‐guided OECs were observed to orient and migrate along the MF direction, forming a regenerative track resembling Büngner bands and offering potential for guiding the growth and alignment of regenerating axons in neurons. Nevertheless, as SPIONs have been reported to induce neuronal degeneration in a dose‐dependent manner, careful consideration must be given to SPIONs administration targeting neuronal cells.

EVs are crucial for intercellular communication as they exhibit conserved and bioactive properties. In recent years, their role in regenerative medicine has gained momentum.^[^ ^31^ ^]^ However, the yield of EVs is limited by the poor secretion abilities of donor cells, the complexity and expenses for large‐scale cell culturing, and the time‐consuming, low‐efficiency procedures for EV production.^[^ ^32^ ^]^ In this study, SPIONs were used as a mechanical force generator to direct the movement of OECs under MF. Due to the fundamental mechanosensing pathway inherent in cell‐cell and cell‐environment communication, cells generally exhibit sensitivity to mechanical load possibly affecting EV secretion.^[^ ^33^ ^]^ To date, several studies in vitro have revealed that mechanical shear stress and turbulence, stretch, and compression could promote EV production.^[^ ^34^ ^]^ Guo et al. revealed the increased EV yield by a low shear was mediated by yes‐associated protein (YAP), and the addition of a YAP inhibitor significantly decreased EV secretion.^[^ ^34a^ ^]^ Patel et al. utilized a 3D‐printed scaffold‐perfusion bioreactor system to increase EV production from endothelial cells.^[^ ^35^ ^]^ Moreover, our previous study has confirmed that the secretion and composition of EVs are sensitive to mechanical cues.^[^ ^36^ ^]^ In agreement with previous studies, our results verified that the secretion of EVs from SPIONs‐OECs was markedly enhanced when exposed to an MF, thereby augmenting the transfer efficiency of exogenous miR‐26a from OECs to neurons. To examine the effect of SPIONs on EV biology, we obtained EVs from OECs loaded with SPIONs (without MF). We found that SPIONs alone were not sufficient to affect the yield and morphology of OECs‐EVs. In addition, the miR26a level was not altered in OECs‐EVs loaded with SPIONs vector, indicating that SPIONs alone had a negligible effect on EVs physiology. However, applying MF significantly boosted EV production from SPIONs‐OECs, which suggested that mechanical cues have a more profound effect on the secretion of EVs from OECs. Despite the above findings, more studies were needed to uncover the mechanism underlying the mechanical cues‐mediated EV production.

In our experiment, the synergistic interaction of intracellular SPIONs and MF exerted an effect on OECs, triggering enhanced migration and EV secretion.^[^ ^34^, ^37^ ^]^ The total force exerted on a single OEC was calculated using the following physical equations:

(1) $$F=\left(m\cdot \nabla \right)\;B$$

This expression defines the magnetic force (*F*) acting on the SPIONs with a magnetic moment (*m*), where *B* represents the magnetic flux density of the applied MF and *m* = *VpMp* is the magnetic dipole moment. Here, *Vp* and *Mp* denote the volume of the magnetic nanoparticles (diameter = 76.7 nm) and the particle magnetization per unit volume, respectively. The mass of SPIONs ingested by OECs was found to be 0.31 ± 0.3 pg at T = 23.17 °C, corresponding to several SPIONs per cell or 7.9 × 10^5 SPIONs.^[^ ^30^, ^38^ ^]^ Consequently, a single OEC is subjected to a force (*F_cell_*), proportional to the number of magnetic particles entrapped within OECs:

(2) $$F_{\mathrm{cell}}=n^{\mathrm{SPIONs}/\mathrm{cell}}\cdot F$$

This mechanical load on OECs was shown to be capable of manipulating the SPIONs‐OECs, including directing migration and alignment and enhancing EV production.

EVs’ biological effects stem from the horizontal transfer of active cargo molecules from their producing cell.^[^ ^39^ ^]^ Neonatal umbilical cord mesenchymal stem cells‐derived EVs have demonstrated potential in rejuvenating aged bone marrow‐derived mesenchymal stem cells and mitigating age‐related degeneration via the transfer of PCNA mRNA transcripts.^[^ ^40^ ^]^ Our study found that miR‐26a@SPIONs‐OECs effectively delivered miR‐26a to co‐cultured neurons via OEC‐EVs under MF exposure, significantly enhancing their intrinsic axon growth ability.

Further, several studies have revealed the influence of miR‐26a on neural progenitor differentiation cell‐cycle progression and neurite outgrowth.^[^ ^41^ ^]^ In our study, we found that the positive effect of miR‐26a‐enriched OEC‐EVs on axonal growth mainly resulted from the suppression of the PTEN and GSK‐3β signaling pathways in neurons. Intriguingly, we also found that miR‐26a was capable of inhibiting astrocyte activation, which holds values for reducing glia scar formation to minimize the physical barrier that impedes neural cell growth. Nevertheless, the mechanism underlying the miR‐26a‐mediated effect on astrocytes was not explored, which is a limitation of the present study. MiR‐26a may target the key molecules for astrocyte activation, such as CEBPD (CCAAT/enhancer binding protein) and GALNT15UDP‐N‐acetyl‐alpha‐D‐galactosamine: polypeptide N‐acetylglucosaminyltransferase 15), both of which have potential binding sites for miR‐26a in astrocytes. Therefore, further studies were needed to fully uncover the mystery of miR‐26a in astrocytes.

It has been recognized that mechanical cues resulting from both intracellularly generated and externally applied forces have broad effects on cellular function.^[^ ^42^ ^]^ The pro‐regenerative strategy employed in this study is not limited to OECs but may extend to various types of cells used in clinical settings, including mesenchymal cells derived from bone marrow and umbilical cord stem cells. Stem cells and their local microenvironment niches communicate through mechanical cues to regulate cell fate and behavior and to guide biological processes,^[^ ^42^ ^]^ indicating that the present mechanical stimulation system holds the potential to modulate the biological functions of various stem cells. In addition, stem cells have a wider range of applications in the context of regenerative medicine, making the current system more attractive in promoting tissue regeneration. Furthermore, this method shows promise for drug delivery for treating diseases of other systems such as cardiovascular and locomotor disorders. While miR‐26a was chosen in this study as an exogenous bioactive cue to boost the regenerative capacity of injured neurons, additional bioactive cues that synergistically regulate inflammation in early stages and promote nerve growth in subsequent stages are yet to be explored. Moreover, disease‐specific requirements will necessitate further clarification of the optimal SPIONs concentration and MF intensity.

## 4 Conclusion

In summary, we developed a potentially effective in vivo pro‐regenerative system for treating SCI, which can concurrently enhance the diminished intrinsic regenerative ability of neurons and provide a beneficial axon guidance environment. An exogenous MF can stimulate miR‐26a@SPIONs‐OECs to deliver miR‐26a to damaged neurons via EVs shuttle from OECs to axons. This system can enhance axonal growth in CNS neurons via the miR‐26a/PTEN‐GSK‐3β axis. Additionally, the use of SPIONs as cellular force generators in an MF led to the oriented migration of OECs and increased secretion of miR‐26a‐EVs. The aligned OECs provided a regenerative “track” for axons. Their secreted EVs further bolstered the regenerative ability of injured neurons and alleviated astrocyte activation at the injury site.

## 5 Experimental Section

### Characterization of PEI‐SPIONs

The morphology, distribution, and average size of PEI‐SPIONs were examined using an atomic mechanical microscope and TEM (H‐600; Hitachi, Tokyo, Japan). Using a zeta potential/nanometer particle size analyzer (DelsaNano; Beckman Coulter, Brea, CA, USA), the zeta potential and size distribution of the PEI‐SPIONs were calculated at 20–25 °C.

### Cell Counting Kit 8 (CCK‐8) Assays

According to the manufacturer's instructions, the CCK‐8 test (Dojindo, Kumamoto, Japan) was used to evaluate the viability and cytotoxicity of the cells.^[^ ^43^ ^]^ In 96‐well plates, OEC was magnetofected with PEI‐SPIONs at various doses (0, 0.5, and 1 g mL^−1^). The OECs were washed three times with sterile PBS (HyClone) after being incubated for 24 or 72 h. Then, 100 L of new media containing 10 L of the CCK‐8 reagent was added to each well, and it was incubated for 4 h at 37 °C in an environment that was humidified with 5% carbon dioxide. Utilizing a microplate reader (Synergy H1, BioTek, Vermont, USA), the absorbance was determined at 450 nm.

### LIVE/DEAD Assay

A LIVE/DEAD Cell Imaging Kit (Thermo Fisher Scientific Inc., Wyman Street, Waltham, MA, USA) was used to conduct LIVE/DEAD assays on a 24‐well plate in accordance with the manufacturer's instructions.^[^ ^44^ ^]^ Green and red hues made live and dead cells evident. Briefly, OECs were magnetofected with PEI‐SPIONs at varied concentrations (0, 0.5, and 1 g mL^−1^). The cells were given three PBS (HyClone) washes after being incubated for 24 or 72 h, and then the staining solution was added. After 15 min of incubation at 37 °C, the number of living and dead cells was counted to determine the cell viability. Counting cells that were alive (green) or dead (red) was done using the Image Pro Plus program from Media Cybernetics in Bethesda, Michigan, in the United States.

### Magnetofection of OECs

The PEI‐SPIONs and miR‐26a plasmids were mixed vigorously after the addition of 1 liter of the PEI‐SPIONs solution (0.5 g L^−1^) to 200 liters of the diluted plasmid solution. The miR‐26a@SPIONs complexes were created by mixing microRNA with magnetic nanoparticles and preparing them at room temperature for 30 min. In a 6‐well plate filled with DMEM/F12 (HyClone) containing 15% FBS, primary OECs were cultivated until they were 70%–80% confluent. Before transfection, cells were washed twice with sterile PBS (HyClone) or DMEM/F12 (HyClone) without serum. The media was then changed with 1.8 mL of brand‐new medium free of serum per well. The prepared miR‐26a@SPIONs complex or NC@SPIONs complex solution was then applied to each well of the 6‐well cell culture plate in a volume of 200 L. After mixing, the 6‐well cell culture plate was put on top of a 1.4 T‐strong, commercial magnetic neodymium–iron–boron multiwell plate (MagnetoFACTOR‐96 plate; Chemical) for 4 h of incubation at 37 °C in a 5% carbon dioxide environment. The MagnetoFACTOR‐96 plates' unique design and alternating north and south polarity guarantee that the MF strength in each well is constant and equal. The magnetofected OECs with miR‐26a@SPIONs or NC@SPIONs complexes were then grown for further usage after the medium was replenished with new serum containing 15% FBS.

### Electron Microscopic Analysis of OECs after Magnetofection with PEI‐SPIONs

To examine the morphology of primary OECs following magnetofection, they were plated on a slide covered with Hyaluronic acid Hydrogel (Sigma–Aldrich) and magnetofected with PEI‐SPIONs (0.5 g mL^−1^). Following magnetofection, the cells were washed with sterile PBS (HyClone) at 6 h to eliminate any dead or detached cells, and then serially fixed and dehydrated with ethanol solution. The cells were then vacuum‐dried at room temperature, gold‐sputtered onto them, and the samples were examined using a scanning electron microscope (S‐3400N; Hitachi). To check for the presence of PEI‐SPIONs in OECs, primary OECs were sown in three wells of the 6‐well plate and treated with PEI‐SPIONs (2 g mL^−1^). The cells were treated in accordance with the industry‐standard TEM imaging (H‐600; Hitachi) methodology after being grown for 24 h.

### Gene Expression Analysis Through Quantitative Real‐Time Polymerase Chain Reaction (qRT‐PCR)

Following the manufacturer's instructions, total RNA was extracted using the TRIzol reagent (Invitrogen, Carlsbad, CA, USA) from OECs magnetofected with PEI‐SPIONs at various safety concentrations (1 and 2 g mL^−1^) after 72 h. Total RNA was then extracted and adjusted to the number of cells. To determine the expression of miR‐26a, cDNA was created using a miRCURY LNA RT Kit from Qiagen (Germany), and qRT‐PCR was carried out using a miRCURY LNA SYBR Green PCR Kit.

### Western Blotting Analysis

Western blotting was performed as previously described.^[^ ^31a^ ^]^ OECs or OECs magnetofected with miR‐26a@SPIONs or NC@SPIONs were seeded in 6‐well plates at 72 h, washed twice with PBS (HyClone), and lysed with radioimmunoprecipitation assay buffer, which contains 1 mM phenylmethanesulfonyl fluoride. By scraping the plates, the lysates were removed, and they were then centrifuged at 10 000 g for 5 min at 4 °C. The bicinchoninic acid (Beyotime Institute of Biotechnology, Shanghai, China) test was used to calculate the total protein concentration. In a nutshell, 30 L of total proteins were electrophoresed individually in 10% sodium dodecyl sulfate‐polyacrylamide gel electrophoresis acid gel and then transferred at 0.8 mA cm^−2^ for 2 h onto a polyvinylidene difluoride membrane. After blocking with a blocking solution (5% skim milk in Tris‐buffered solution plus Tween‐20: 50 mM Tris‐hydrochloride, 150 mM NaCl, pH = 7.5, 0.1% volume/volume Tween 20) at room temperature for 2 h, the membranes were incubated at 4 °C overnight with mouse anti‐PTEN antibody at 1:1000 (Novus Biologicals, CO, USA) and rabbit anti‐β‐III‐tubulin monoclonal antibody at 1:3000 (Cat. 2128; CST, Boston, MA, USA). The membranes were treated with a secondary antibody that was horseradish peroxidase‐conjugated after being washed twice in Tris‐buffered solution + Tween‐20 for 10 min. Then, PBS (HyClone) was used three times for 5 min of washing. The membranes were evaluated using the GE AI600 equipment (General Electric Company, Boston, MA, USA) and ClarityTM Western ECL Substrate (BIO‐RAD, CA, USA) to look for immunoreactive proteins. GAPDH was used as an internal control.

### DRG Neurons Co‐Culture with OECs in Separate

The Transwell system to achieve a separate culture of two kinds of cells to elucidate the influence of miR‐26a@SPIONs‐OECs on the neurite outgrowth of DRG neurons was utilized. Briefly, DRG neurons were isolated and inoculated on 6‐well plates that had been pre‐coated to allow cell adherence. The OECs were treated in different ways and inoculated in the upper chamber of the Transwell Petri dish(8.0 µm) arranged in the 6‐well plate. The Transwell system was then placed in an incubator at 37 °C with 5% CO_2_ for 48 h.

### In Situ Hybridization

The OECs were magnetically transfected with SPIONs and miR‐26a after being infected in a 6‐well plate and covered in advance with square crawling slides. After 24 h of aspiration, the medium was fixed with 4% paraformaldehyde; the miR‐26a probe was then sealed with sealing film and placed on a floating plate centrifuge tube rack; it was then warmed in a constant temperature water bath at 75 °C for 5 min; ice was then made; and the centrifuge tube was transported to a refrigerator at 0 °C for 5–10 min to promote denaturation of double‐stranded DNA probes.^[^ ^31b^ ^]^ The prepared slide specimens were baked in an incubator at 55 °C for two to 3 h, and then submerged for two to 3 min in a modified solution of formamide/2 at 70 °C or 75 °C with a volume fraction of ≈70%. The specimens were then sequentially dehydrated for 5 min using 70% ice ethanol, 90% ice ethanol body, and 100% ice ethanol. Finally, they were allowed to air dry. The denatured and dehydrated slide specimen was inverted at the position of the DNA probe, the edge of the slide was glued with nail polish, and the slide was then put in a moist box for overnight hybridization at 37° C (≈15–17 h); The hybridized slide sample was submerged in a mixture of 50% formamide and 2% SSC (pre The slide specimens were cleaned three times in 50% formamide/2 SSC (heated to 42–50 °C) for 5 min each, and then three times in 1 SSC for 5 min each. The slides were removed from the slide specimens and allowed to dry naturally before the re‐staining solution (200 L of PI/antifade staining solution or DAPI/antifade staining solution) was added dropwise. The coverslips were then placed over the slide specimens, and the slides were then sealed with a sealing solution. To completely shut and set the slides, nail polish was smeared around the coverslips and used as a sealant. The miR‐26a in the cells was then selectively tagged in green, and these observations were made using a fluorescence microscope.

### Immunofluorescence Staining

DRG neurons were extracted and inoculated on a 6‐well plate encapsulated in advance, and after 12 h of apposition, the upper chamber of the Transwell culture dish was placed in the 6‐well plate. The OECs were digested after 24 h of magnetic transfection with SPIONs and inoculated in the upper chamber and incubated in an incubator at 37 °C and 5% CO_2_ for 48 h. The staining was performed according to the conventional immunofluorescence staining operation steps: sterile PBS wash, 4% paraformaldehyde was fixed for 15–20 min, and 2% Triton was used to punch the cells for 10 min to wash them respectively. Goat serum was closed for 1 h, the primary antibody was added overnight at 4°, and washed and secondary antibody was added, washed, and sealed with anti‐fluorescence quenching sealer containing DAPI. This was then observed under a confocal microscope and photographed.

The DRG neurons were stained with the β‐III‐tubulin (1:200, Abcam) antibody and the DRG explants were stained with β‐III‐tubulin (1:200, Abcam) and S100 (1:500, Abcam) antibodies. The OEC was stained with SMA (1:200, Abcam) and p75^NTR^ (1:200, Abcam) antibodies. The astrocyte was stained with the GFAP (1:400, Abcam) antibody.

### EVs Isolation

EVs (diameter > 100 nm) were isolated and purified by gradient centrifugation processes, as it was described previously.^[^ ^45^ ^]^ First, the primary OECs were cultured and purified following the previous protocol,^[^ ^46^ ^]^ and were seeded at a density of 1.8 × 10^4^ cells cm^−2^ with a complete medium containing 10% EVs‐depleted FBS for 48 h. Cell viability was assessed using a CCK‐8 kit, and only medium from cultures with > 90% viability and the primary OECs at the third passage (P3) were used for EVs harvesting, which showed a high purity (> 95%). Medium of different groups of OECs was harvested and centrifuged at 750 g for 20 min to remove cells and subsequently centrifuged at 2000 g for 30 min to remove cell debris and apoptotic bodies. Then, the supernatant was centrifuged at 16 000 g for 70 min and the obtained EV pellets were resuspended with pre‐cold PBS followed by centrifugation at 16 000 g for 70 min. All the centrifugation procedures were performed under 4 °C, and the final EVs pellets were resuspended in 100.0 µL of pre‐cold PBS and stored at −80 °C. Second, NTA results showed the size of the obtained EVs within a stable range (182.4 ± 1.70 nm), indicating they are homogeneous. The concentration of EVs was assayed by NTA to be (3.15±0.68) × 10^9^/flask.

The EVs were characterized by TEM (TECNAI Spirit, FEI, USA) and Western blotting analysis. NTA (ZetaView, Particle Metrix, Germany) was also performed to measure the concentration and size distribution of EVs. The NTA and BCA analysis (Beyotime Biotechnology, Shanghai, China) were performed to quantify the obtained EVs. In the Western blotting analysis to identify the EV markers, 2 µg of SCs lysate and 2 µg of SCs‐EVs were loaded in each lane.

### DRG Neurons/Explants Co‐Cultured with OECs

It was first magnetofected OECs with SPIONs or miR‐26a@SPIONs. The magnet was positioned parallel to the edge of the culture plate 15 cm away after the cells adhered. After being treated with the MF for 48 h, the miR‐26a@SPIONs‐OECs and SPIONs‐OECs exhibited excellent alignment. Then, the DRG neurons and explants were seeded on the aligned OECs layer and incubated at 37 °C and 5% CO_2_ to observe the axonal growth of DRG neurons and explants.

### Inverted Coverslip Migration Test

The inverted coverslip assay was performed as previously described.^[^ ^44^, ^47^ ^]^ OECs that had undergone different treatments were resuspended at a density of 2 × 10^6^ cells mL^−1^, inoculated onto square slides of 24‐well plates covered with matrix gel, and then incubated for 24–36 h until OECs had adhered to the surface. They were then cleaned with sterile PBS (HyClone) and put upside‐down into plates with glass bottoms that were lined with astrocytes for cell culture. For two days, astrocyte density was 1 × 10^5^ to allow for cell migration in the DMEM/F12 complete media. For further testing, a cubic magnet with a cubic side length of 50 mm and an MF strength of 1.4 T was positioned parallel to the edge of the coverslip. Cells were then fixed, and immunofluorescence staining was carried out. The greatest distance and quantity of migrating OECs were measured and recorded. The distance at which the cells left the square slide was examined. Additionally, the angle in the outward MF direction and the angle between the long axis of each migrating OEC and the marker were measured. Oi = cos(θ) with angles of 0, 1, and 2 (Oi = 1 when the OEC migration direction coincides with the MF direction, and Oi = 0.5 or 0 when the angle between the OEC migration direction and the MF direction is 60° or 90°) were used to define the direction index, which represents the direction representing OEC migration. The migration angle is more likely to be in the MF direction when Oi is closer to 1.

### OEC and Astrocyte Confrontation Assays

The OEC and astrocyte countermeasure assays were performed as previously described with some modifications.^[^ ^48^ ^]^ In brief, some modifications were made as previously described. Astrocytes (density: 6 × 10^5^ mL^−1^) or OECs (density: 2 × 10^6^ mL^−1^) were inoculated in parallel with each other on a confocal dish coated with stromal gel. After 3 h of attachment, cells were washed with sterile PBS (HyClone) and MFs were applied in parallel with OECs on the astrocyte side. The cells were cultured using DMEM/F12 complete medium for 7 days and migrated toward each other. Subsequently, the cells were fixed, and immunofluorescence staining was performed. SCs that crossed the cell border and migrated to the astrocytic region were counted and more than six regions were randomly selected in each region.

### SD Rat Spinal Cord Injury Hemisection Modeling

All animal experimental procedures were performed following the Guide for the Care and Use of Laboratory Animals (National Institutes of Health Publication No 80‐23, revised 1996) and approved by the Animal Research Committee of The Fourth Military Medical University, People's Republic of China (NO. IACUC‐20230043). The animals were housed at a constant temperature (22–24°C) and maintained in sawdust‐lined plastic cages under a 12 h light–dark cycle with free access to laboratory chow pellets and tap water. Healthy adult male Sprague–Dawley (SD) rats, weighing 220−240 g (provided by Laboratory Animal Center of the Fourth Military Medical University). The rats were injected with sodium pentobarbital intraperitoneal anesthesia and fixed in a prone position, and the back hair was removed with a shaver and disinfected with an iodophor.^[^ ^49^ ^]^ The right half of the spinal cord was transected with microsurgical scissors to the right edge of the median spinal vein, without injuring the vein, and six different groups were created: 1) The sham group, operated mice without SCI; 2) The CONT group, the untreated SCI rats; 3) The SPIONs‐OECs group, the SCI rats treated with SPIONs‐OECs; 4) The SPIONs‐OECs+MF group, the SCI rats treated with both SPIONs‐OECs and MF; 5) The miR‐26a@SPIONs‐OECs group, the SCI rats treated with miR‐26a@SPIONs‐OECs; 6) The miR‐26a@SPIONs‐OECs +MF group, the SCI rats treated with both miR‐26a@SPIONs‐OECs and MF. It was implanted 5 × 10^5^ mL^−1^ miR‐26a@SPIONs‐OECs into the lesion site using Hyaluronic acid hydrogel, then exposed them to an MF (1.4T, 2 h day^−1^ for 8 weeks). Finally, the muscle and skin were sutured with sterile band sutures and cefuroxime was injected intraperitoneally after the operation and awakened naturally at room temperature.

### BBB Score

The BBB scale was an internationally recognized index for assessing the functional repair process of human spinal cord injury,^[^ ^50^ ^]^ with a total of twenty‐one points, and consists of the following three stages: i) the earliest stage was characterized by the almost complete absence of front and hind limb joint exercise; ii) the middle stage was characterized by a small amount of ataxic gait; iii) the late stage was characterized by the observation of fine motor processes, such as the inability of the paws and tail to strongly support pulling and dragging walking, as well as trunk imbalance and alternate rotation of the paws. Three or more researchers (it was not clear what exactly happened in the experiment), each independently completed the scoring, once a week, and statistical analysis.

### Immunofluorescence Assay

Neural paraffin sections were used to detect nerve regeneration by immunofluorescent staining. After de‐paraffinization and rehydration, sections were subjected to antigen retrieval using microwave thermal repair followed by staining. Briefly, sections were blocked with 1% BSA for 30 min at 24 °C. The blocking solution was removed and the primary antibody working solution was added and needed to completely cover the sections. The sections were placed in a humidified chamber overnight at 4 °C. The sections were then washed 3 times with PBS for 3 min each. After washing with PBS, the samples were completely covered with a secondary antibody working solution. Incubate for 1 h at 37 °C in the dark. After incubation with DAPI working solution (10 min in the dark at room temperature), wash with PBS and photographed with laser scanning confocal microscopy (LSCM, NIKON). Primary antibodies Anti‐GFAP (1:400, Abcam), Anti‐GAP43(1:200, Abcam), incubated with secondary antibodies: indocarbocyanine‐conjugated goat anti‐chicken (1:500; Beijing Com Win Biotech Co., Ltd.) or indocarbocyanine‐conjugated goat anti‐mouse (1:200; Beijing Com Win Biotech Co., Ltd.)

### Motor Evoked Potentials Evaluation

Using an Electromyograph and Evoked Potential Equipment (33A07) from Dantec Dynamics, Copenhagen, Denmark, motor evoked potentials (MEPs) were assessed. Pentobarbital sodium solution (30 mg k^−1^ g) at a concentration of 1% (w/v) was administered intraperitoneally to anesthetize rats. The MEP was based on the stimulation of the corticospinal tract, which emerges from the motor cortex, by a transcutaneous bipolar electrode positioned at the motor cortex's surface on the skull. Recording electrodes were subcutaneously placed into the tibialis anterior muscle. A grounding electrode was subcutaneously implanted into the back. Applied was a single stimulus of 50 mA.

### VonFrey Test and Hotplate Test

The paws of the rats were stimulated using the Von Frey Tactile Pain Apparatus BIO‐EVF3 (Bioseb, Shanghai huanxi) and sufficient time intervals were left after each stimulation to allow the animals to return to the basal state, the animals were observed to respond to different stimuli, such as lifting their feet or making sounds, to determine their pain sensitivity, the test was repeated several times, and record the results of each test. The hot and cold plate pain meter LE7420 (Bioseb, Shanghai huanxi) was set at 50 °C and the rats were placed on the hot and cold plate so that their paws touched the surface of the platform. The time of each response was recorded, usually using seconds as the unit, and for thermal stimuli, the time the animal licked its paw was usually recorded. If the animal does not respond within 30 s, this should be recorded as 30 s.

### Statistical Analysis

All statistical analyses were performed using GraphPad Prism 9.0 (GraphPad Software, Inc., USA). Data were shown as mean ± SD (Standard Deviation). Data were analyzed using Student's t‐test or one‐way analysis of variance (ANOVA) followed by Dunnett's multiple comparisons test. The values were considered significantly different at *P* < 0.05.

## Conflict of Interest

The authors declare no conflict of interest.

## Supporting information

Supporting Information

Supplemental Video 1

Supplemental Video 2

## Data Availability

The data that support the findings of this study are available in the supplementary material of this article.

## References

[1] H. Huang , W. Young , S. Skaper , L. Chen , G. Moviglia , H. Saberi , Z. Al‐Zoubi , H. S. Sharma , D. Muresanu , A. Sharma , W. El Masry , S. Feng , J. Orthop. Translat. 2020, 20, 14. 31908929 10.1016/j.jot.2019.10.006 PMC6939117

[2] J. E. Frontera , P. Mollett , Phys. Med. Rehabil. Clin. N. Am. 2017, 28, 821. 29031346 10.1016/j.pmr.2017.06.013

[3] J. H. Badhiwala , C. S. Ahuja , M. G. Fehlings , J Neurosurg. Spine 2018, 30, 1. 30611186 10.3171/2018.9.SPINE18682

[4] a) W. Xue , W. Shi , Y. Kong , M. Kuss , B. Duan , Bioact. Mater. 2021, 6, 4141. 33997498 10.1016/j.bioactmat.2021.04.019 PMC8099454

[5] a) M. Karsy , G. Hawryluk , Curr. Neurol. Neurosci. Rep. 2019, 19, 65. 31363857 10.1007/s11910-019-0984-1

[6] a) F. Barnabé‐Heider , J. Frisén , Cell Stem Cell 2008, 3, 16. 18593555 10.1016/j.stem.2008.06.011

[7] Z. Cheng , W. Zhu , K. Cao , F. Wu , J. Li , G. Wang , H. Li , M. Lu , Y. Ren , X. He , Int. J. Mol. Sci. 2016, 17, 1380. 27563878 10.3390/ijms17091380 PMC5037660

[8] A. D. Gilmour , R. Reshamwala , A. A. Wright , J. A. K. Ekberg , J. A. St John , J. Neurotrauma. 2020, 37, 817. 32056492 10.1089/neu.2019.6939

[9] a) H. Fan , Z. Chen , H. B. Tang , L. Q. Shan , Z. Y. Chen , X. H. Wang , D. G. Huang , S. C. Liu , X. Chen , H. Yang , D. Hao , Bioeng. Transl. Med. 2022, 7, e10287. 35600663 10.1002/btm2.10287 PMC9115713

[10] X. Wang , C. Jiang , Y. Zhang , Z. Chen , H. Fan , Y. Zhang , Z. Wang , F. Tian , J. Li , H. Yang , D. Hao , Cell Biosci. 2022, 12, 23. 35246244 10.1186/s13578-022-00765-y PMC8895872

[11] a) P. Assinck , G. J. Duncan , B. J. Hilton , J. R. Plemel , W. Tetzlaff , Nat. Neurosci. 2017, 20, 637. 28440805 10.1038/nn.4541

[12] R. M. Gómez , M. Y. Sánchez , M. Portela‐Lomba , K. Ghotme , G. E. Barreto , J. Sierra , M. T. Moreno‐Flores , Glia 2018, 66, 1267. 29330870 10.1002/glia.23282

[13] R. G. Rowe , J. Mandelbaum , L. I. Zon , G. Q. Daley , Cell Stem Cell 2016, 18, 707. 27257760 10.1016/j.stem.2016.05.016 PMC4911194

[14] K. K. Park , K. Liu , Y. Hu , P. D. Smith , C. Wang , B. Cai , B. Xu , L. Connolly , I. Kramvis , M. Sahin , Z. He , Science 2008, 322, 963. 18988856 10.1126/science.1161566 PMC2652400

[15] a) C. Lucci , R. Mesquita‐Ribeiro , A. Rathbone , F. Dajas‐Bailador , Development 2020, 147, 180232. 10.1242/dev.180232 PMC7033742 31964775

[16] A. I. Faden , J. Wu , B. A. Stoica , D. J. Loane , Br. J. Pharmacol. 2016, 173, 681. 25939377 10.1111/bph.13179 PMC4742301

[17] M. Mahar , V. Cavalli , Nat. Rev. Neurosci. 2018, 19, 323. 29666508 10.1038/s41583-018-0001-8 PMC5987780

[18] S. Guo , N. Perets , O. Betzer , S. Ben‐Shaul , A. Sheinin , I. Michaelevski , R. Popovtzer , D. Offen , S. Levenberg , ACS Nano 2019, 13, 10015. 31454225 10.1021/acsnano.9b01892

[19] C. Radtke , J. D. Kocsis , Cells Tissues Organs. 2014, 200, 48. 25765445 10.1159/000369006

[20] B. Xia , J. Gao , S. Li , L. Huang , T. Ma , L. Zhao , Y. Yang , J. Huang , Z. Luo , Front. Cell Neurosci. 2019, 13, 548. 31866834 10.3389/fncel.2019.00548 PMC6908849

[21] P. N. Rizek , M. D. Kawaja , Neuroreport 2006, 17, 459. 16543806 10.1097/01.wnr.0000209000.32857.1b

[22] A. Jahed , J. W. Rowland , T. Mcdonald , J. G. Boyd , R. Doucette , M. D. Kawaja , J. Comp. Neurol. 2007, 503, 209. 17492622 10.1002/cne.21385

[23] C. S. Ahuja , A. Mothe , M. Khazaei , J. H. Badhiwala , E. A. Gilbert , D. Kooy , C. M. Morshead , C. Tator , M. G. Fehlings , Stem Cells Transl. Med. 2020, 9, 1509. 32691994 10.1002/sctm.19-0135 PMC7695641

[24] Y. Iwashita , J. W. Fawcett , A. J. Crang , R. J. M. Franklin , W. F. Blakemore , Exp. Neurol. 2000, 164, 292. 10915568 10.1006/exnr.2000.7440

[25] H. Kanno , Y. Pressman , A. Moody , R. Berg , E. M. Muir , J. H. Rogers , H. Ozawa , E. Itoi , D. D. Pearse , M. B. Bunge , J. Neurosci. 2014, 34, 1838. 24478364 10.1523/JNEUROSCI.2661-13.2014 PMC3905147

[26] K. I. Lee , M. Olmer , J. Baek , D. D. D'lima , M. K. Lotz , Acta Biomater. 2018, 76, 126. 29908335 10.1016/j.actbio.2018.06.021 PMC6090559

[27] L. Kong , X. Gao , Y. Qian , W. Sun , Z. You , C. Fan , Theranostics 2022, 12, 4993. 35836812 10.7150/thno.74571 PMC9274750

[28] A. Avasthi , C. Caro , E. Pozo‐Torres , M. P. Leal , M. L. García‐Martín , Top. Curr. Chem. (Cham) 2020, 378, 40. 32382832 10.1007/s41061-020-00302-w PMC8203530

[29] a) S. S. Eamegdool , M. W. Weible , B. T. T. Pham , B. S. Hawkett , S. M. Grieve , T. Chan‐Ling , Biomaterials 2014, 35, 5549. 24726535 10.1016/j.biomaterials.2014.03.061

[30] C. Riggio , M. P. Calatayud , M. Giannaccini , B. Sanz , T. E. Torres , R. Fernández‐Pacheco , A. Ripoli , M. R. Ibarra , L. Dente , A. Cuschieri , G. F. Goya , V. Raffa , Nanomed.: Nanotech., Bio. Medic. 2014, 10, 1549. 10.1016/j.nano.2013.12.008 24407149

[31] a) Y. Zhang , I. Naguro , A. E. Herr , Angew. Chem. 2019, 58, 13929. 31390130 10.1002/anie.201906920 PMC6759404

[32] J. J. Lai , Z. L. Chau , S.‐Y. Chen , J. J. Hill , K. V. Korpany , N.‐W. Liang , L.‐H. Lin , Y.‐H. Lin , J. K. Liu , Y.‐C. Liu , R. Lunde , W.‐T. Shen , Adv. Sci. (Weinh) 2022, 9, e2103222. 35332686 10.1002/advs.202103222 PMC9130923

[33] Q. Zhu , M. Heon , Z. Zhao , M. He , Lab Chip 2018, 18, 1690. 29780982 10.1039/c8lc00246k PMC5997967

[34] a) S. Guo , L. Debbi , B. Zohar , R. Samuel , R. S. Arzi , A. I. Fried , T. Carmon , D. Shevach , I. Redenski , I. Schlachet , A. Sosnik , S. Levenberg , Nano Lett. 2021, 21, 2497. 33709717 10.1021/acs.nanolett.0c04834

[35] D. B. Patel , C. R. Luthers , M. J. Lerman , J. P. Fisher , S. M. Jay , Acta Biomater. 2019, 95, 236. 30471476 10.1016/j.actbio.2018.11.024 PMC6531369

[36] B. Xia , J. Gao , S. Li , L. Huang , L. Zhu , T. Ma , L. Zhao , Y. Yang , K. Luo , X. Shi , L. Mei , H. Zhang , Y. Zheng , L. Lu , Z. Luo , J. Huang , Theranostics 2020, 10, 8974. 32802175 10.7150/thno.44912 PMC7415818

[37] A. Pinto , I. Marangon , J. Méreaux , A. Nicolás‐Boluda , G. Lavieu , C. Wilhelm , L. Sarda‐Mantel , A. K. A. Silva , M. Pocard , F. Gazeau , ACS Nano 2021, 15, 3251. 33481565 10.1021/acsnano.0c09938

[38] J. N. Fass , D. J. Odde , Biophys. J. 2003, 85, 623. 12829516 10.1016/S0006-3495(03)74506-8 PMC1303117

[39] a) R. Kalluri , V. S. LeBleu , Science 2020, 367, eaau6977; 32029601 10.1126/science.aau6977 PMC7717626

[40] Q. Lei , F. Gao , T. Liu , W. Ren , L. Chen , Y. Cao , W. Chen , S. Guo , Q. Zhang , W. Chen , H. Wang , Z. Chen , Q. Li , Y. Hu , A. Y. Guo , Sci. Transl. Med. 2021, 13, eaaz8697. 33504653 10.1126/scitranslmed.aaz8697

[41] K. Attoff , Y. Johansson , A. Cediel‐Ulloa , J. Lundqvist , R. Gupta , F. Caiment , A. Gliga , A. Forsby , Sci. Rep. 2020, 10, 16714. 33028897 10.1038/s41598-020-73698-6 PMC7541504

[42] K. H. Vining , D. J. Mooney , Nat. Rev. Mol. Cell Biol. 2017, 18, 728. 29115301 10.1038/nrm.2017.108 PMC5803560

[43] J. G. Boyd , V. Skihar , M. Kawaja , R. Doucette , Anat. Rec. B New Anat 2003, 271, 49. 12619086 10.1002/ar.b.10011

[44] F. T. Afshari , J. W. Fawcett , Methods Mol. Biol. 2012, 814, 381. 22144320 10.1007/978-1-61779-452-0_25

[45] C. Théry , K. W. Witwer , E. Aikawa , M. J. Alcaraz , J. D. Anderson , R. Andriantsitohaina , A. Antoniou , T. Arab , F. Archer , G. K. Atkin‐Smith , D. C. Ayre , J. M. Bach , D. Bachurski , H. Baharvand , L. Balaj , S. Baldacchino , N. N. Bauer , A. A. Baxter , M. Bebawy , C. Beckham , A. Bedina Zavec , A. Benmoussa , A. C. Berardi , P. Bergese , E. Bielska , C. Blenkiron , S. Bobis‐Wozowicz , E. Boilard , W. Boireau , A. Bongiovanni , et al., J. Extracellular Vesicles 2018, 7, 1535750. 30637094 10.1080/20013078.2018.1535750 PMC6322352

[46] J. R. Higginson , S. C. Barnett , Exp. Neurol. 2011, 229, 2.20816825

[47] P. O'neill , S. L. Lindsay , A. Pantiru , S. E. Guimond , N. Fagoe , J. Verhaagen , J. E. Turnbull , J. S. Riddell , S. C. Barnett , Glia 2017, 65, 19. 27535874 10.1002/glia.23047 PMC5244676

[48] L. Cao , Y.‐L. Zhu , Z. Su , B. Lv , Z. Huang , L. Mu , C. He , Glia 2007, 55, 897. 17405147 10.1002/glia.20511

[49] M. Patel , Y. Li , J. Anderson , S. Castro‐Pedrido , R. Skinner , S. Lei , Z. Finkel , B. Rodriguez , F. Esteban , K.‐B. Lee , Y. L. Lyu , L. Cai , Mol. Ther. 2021, 29, 2469. 33895323 10.1016/j.ymthe.2021.04.027 PMC8353206

[50] Y. Chen , L. Wu , M. Shi , D. Zeng , R. Hu , X. Wu , S. Han , K. He , H. Xu , X. Shao , R. Ma , Frontie. Immunol. 2022, 13, 788556. 10.3389/fimmu.2022.788556 PMC8987202 35401582

