# Correction to “A Novel Magnetic Responsive miR‐26a@SPIONs‐OECs for Spinal Cord Injury: Triggering Neural Regeneration Program and Orienting Axon Guidance in Inhibitory Astrocytic Environment”

**DOI:** 10.1002/advs.76206

**Published:** 2026-07-06

Gao X, Li S, Yang Y, Yang S, Yu B, Zhu Z, Ma T, Zheng Y, Wei B, Hao Y, Wu H, Zhang Y, Guo L, Gao X, Wei Y, Xue B, Li J, Feng X, Lu L, Xia B, Huang J, “A Novel Magnetic Responsive miR‐26a@SPIONs‐OECs for Spinal Cord Injury: Triggering Neural Regeneration Program and Orienting Axon Guidance in Inhibitory Astrocytic Environment,” *Adv* *anced* *Sci* *ence* 10 no. 32, (2023): e2304487, https://doi.org/10.1002/advs.202304487.

In the original published paper, we found that the Western blot images of the “GSK‐3β” and “GAPDH” in Figure 3G were improperly used during sub‐figure assembly. The corrected figure is presented below. Upon meticulous review of the original records, we are confident that the correction does not impact the overall findings and conclusions of this paper.

Corrected Figure 3G:


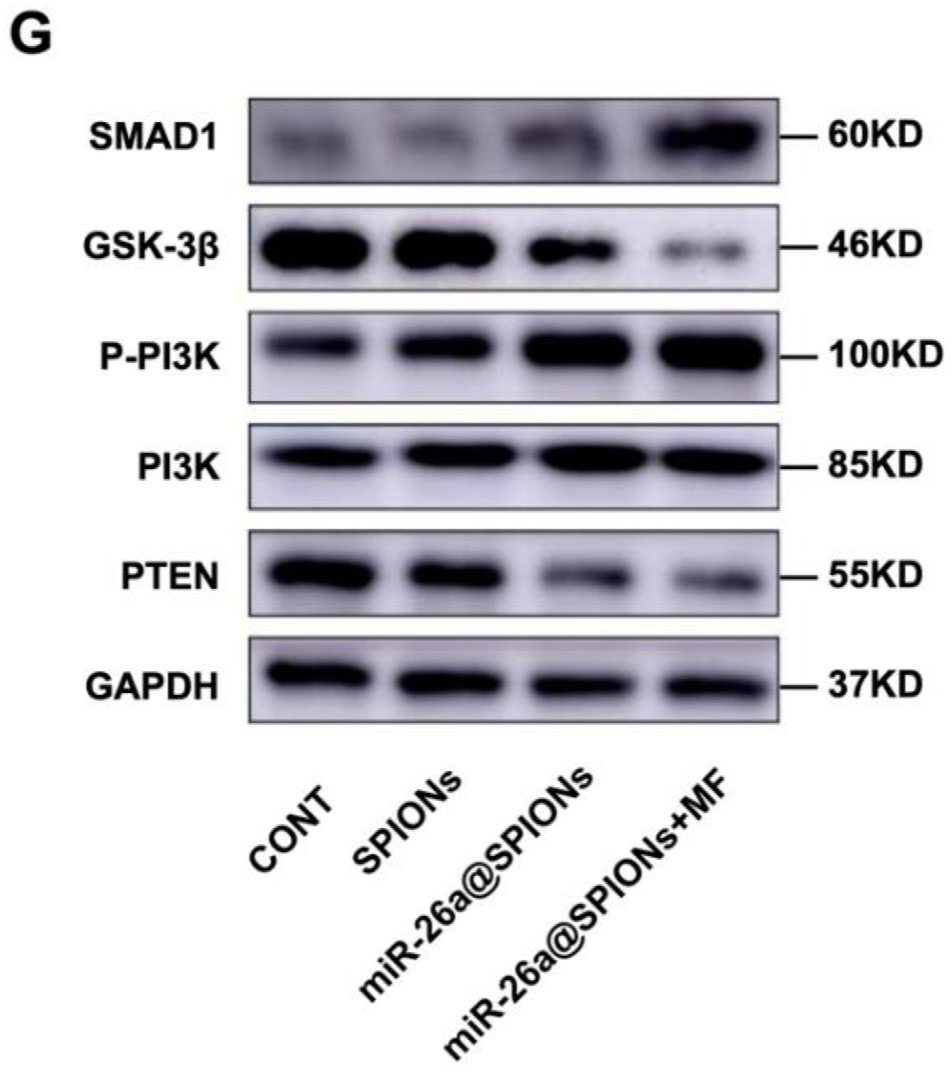


We apologize for this error.

