# Supplementary material for: A Novel Magnetic Responsive miR‐26a@SPIONs‐OECs for Spinal Cord Injury: Triggering Neural Regeneration Program and Orienting Axon Guidance in Inhibitory Astrocytic Environment
Source: Adv Sci (Weinh). 2023 Oct 3;10(32):2304487. doi: 10.1002/advs.202304487 (PMC10646239; doi:10.1002/advs.202304487)
Supplement: Supplementary file 1 — Supporting Information [file ADVS-10-2304487-s002.pdf]

ADVANCED SCIENCE
Open Access

Supporting Information

for *Adv. Sci.*, DOI 10.1002/advs.202304487

A Novel Magnetic Responsive miR-26a@SPIONs-OECs for Spinal Cord Injury: Triggering Neural Regeneration Program and Orienting Axon Guidance in Inhibitory Astrocytic Environment

Xue Gao, Shengyou Li, Yujie Yang, Shijie Yang, Beibei Yu, Zhijie Zhu, Teng Ma, Yi Zheng, Bin Wei, Yiming Hao, Haining Wu, Yongfeng Zhang, Lingli Guo, Xueli Gao, Yitao Wei, Borui Xue, Jianzhong Li, Xue Feng, Lei Lu*, Bing Xia* and Jinghui Huang**

Supporting Information

A Novel Magnetic Responsive miR-26a@SPIONs-OECs for Spinal Cord Injury: Triggering Neural Regeneration Program and Orienting Axon Guidance in Inhibitory Astrocytic Environment

Xue Gao, Shengyou Li, Yujie Yang, Shijie Yang, Beibei Yu, Zhijie Zhu, Teng Ma, Yi Zheng, Bing Wei, Yiming Hao, Haining Wu, Yongfeng Zhang, Lingli Guo, Xueli Gao, Yitao Wei, Borui Xue, Jianzhong Li, Xue Feng, Lei Lu*, Bing Xia*, Jinghui Huang**

Extraction and culture of primary cell:

Culture and Purification of OECs: Primary OECs were isolated from the olfactory bulb of Sprague Dawley rats (postnatal days 1–3; n = 100, provided by the Laboratory Animal Center of the Fourth Military Medical University [FMMU], Xi'an, China) as previously described with some modifications.[1] All experimental procedures were performed under the Guide for the Care and Use of Laboratory Animals and approved by the Animal Research Committee of The Fourth Military Medical University (Xi'an, China). The membrane and blood were stripped before cutting the explant into small pieces, and dissociated with 0.2% of type IV collagenase and 0.25% trypsin for 30 mins at 37 °C, and mixed evenly every 5 mins. Subsequently, the cells were maintained in Dulbecco's Modified Eagle Medium: Nutrient Mixture F-12 (DMEM/F12, HyClone, Logan, Utah, USA) containing 15% fetal bovine serum (FBS; Gibco, Grand Island, State of New York, USA) and 1% antibiotics (penicillin and streptomycin solution). At 24 h after inoculation, replace 1/3 of the medium, at 48 h replace half of the medium, and change all the medium at 72 h. These cultures were highly enriched in OECs (> 95%) based on the immunostaining of the OECs marker SMA and p75 neurotrophin receptor($p75^{NTR}$).

Culture and Purification of Astrocytes: Primary astrocytes were prepared from the cerebral cortices of Sprague Dawley rats (postnatal days 1–3; n = 50, provided by the Laboratory Animal Center of the FMMU) as previously described with some modifications.[2] Take 1-3 days SD rat pups and soak them in 75% ice alcohol for 3-5 min, remove the pups, absorb the excess alcohol with cotton balls, and cut off the head; peel off the cerebral cortex under a stereomicroscope, put them into pre-chilled serum-free DF12, remove the meninges and blood vessels with microforceps, and cut them with curved ophthalmic scissors. Add 0.25% trypsin and type IV collagenase, and incubate the digestion for 30 min at 37 °C in a 5% CO_2 incubator, shaking every 5 min. Digestion was terminated by the gentle blowing of resuspended cells,

followed by inoculation and incubation in a 37 °C, 5% CO_2 incubator; after 10-12 days of incubation, the cells were shaken on a shaker for 24 h at 200 rpm (to remove oligodendrocytes and microglia), purified and passed to 3-4 generations for subsequent experiments.

Culture and Purification of DRG Neurons and Explants: Traditional chemical purification generally relies on several weeks of growth in the presence of antimitotic or cytotoxic substances to target dividing glia cells, which could obtain a neuron purity of around 80-85%.[3] We purified rat dorsal root ganglion neurons according to a previously published immunopanning protocol.[4] Firstly, we coated coverslips with Poly-D-lysine (PDL) and laminin. Briefly, we diluted PDL stock 1:100 in sterile H_2O, covered each coverslip with 100 μL of diluted PDL, and incubated for 30 min at room temperature. After rinsing the coverslips three times with sterile H_2O and drying the coverslips completely, we covered each coverslip with 100 μL of diluted laminin (1mg/ml laminin 1:500 in filtered neurobasal medium) and incubated for 1 d at 37 °C. We prepared the BSL-1 dish (add 20 mL PBS and 20 μL of 5 mg/mL BSL-1 in a 15 cm Petri dish) and the CD9 dish (add 10 mL of 50 mM Tris-HCl (pH 9.5), 30 μL of goat-anti-mouse IgG +IgM (H + L) in a 10 cm Petri dish) and incubated them overnight at 4 °C. Then we washed the BSL1 dish three times with PBS and incubated it with 9mL of 0.2% BSA (diluted with D-PBS) for more than 2 h at room temperature. To the CD9 dish, we washed it three times with PBS and incubated it with 5 mL of 0.2% BSA (diluted with D-PBS) and 15 μL of anti-rat CD9 for at least 2 h at room temperature. Next, we dissect the spine and collect the DRGs. The 1-3 days SD rat pups were taken and soaked in 75% ice alcohol for 3-5 min, microscopic instruments were sterilized in an autoclave, dried in a 65 °C drying oven, and disinfected with UV light for 30 min and placed in an ultra-clean bench. The pups were removed, excess alcohol was absorbed with autoclaved cotton balls, and the head was clipped; blood was absorbed with cotton balls, and skin and bone were cut along the spine, thoracic and cervical vertebrae with microscissors to fully expose the dorsal root ganglia on both sides of the spinal canal. Pick out each small bulbous dorsal root ganglion with very fine ophthalmic forceps carefully peel off the blood, outer membrane, and neurofilaments surrounding the DRG neurons, and cut them up. Add collagenase type IV and 0.25% trypsin to the dish (2 trypsin and 2 collagenase type IV per 5 mice) and transfer to a 15 mL centrifuge tube. During this time, they can be shaken 5-6 times for adequate digestion; add 4 mL of DF-12 medium containing FBS to the tube to terminate the digestion and centrifuge at 1000 rpm for 5 min; resuspend the cell pellet by adding 2 mL panning buffer (18 mL D-BPS + 2 mL of 0.2% BSA + 200 μL of 0.5 mg/mL insulin). The cell suspension was mixed with 6 mL panning buffer, filtered through a 100 μm cell sieve, and washed the filter with 3 mL of panning buffer. We incubated the tube in

a 37 °C, 10% CO_2 incubator for 30 min and loosely cap the tube to allow gas exchange. After rinsing the BSL-1 panning dish three times with D-PBS, we decanted the cell suspension into the BSL-1 dish. We incubated the dish for 10 min at room temperature, gently shaken, and then incubated it for another 10 min (20 min total). Similarly, we rinsed the CD9 dish nine times with D-PBS and transferred the unbound cells from the BSL-1 dish to the CD9 dish. Then the dish was incubated for 15 min at room temperature, gently shaken, and then incubated for another 15 min (30 min total). The suspension of unbound cells was decanted into a 15 mL conical tube and was centrifuged at 1000 rpm for 5 min. Finally, we aspirated the supernatant and resuspended the cell pellet in Warmed Neurobasal medium added NGF (to 100 ng/mL), BDNF (to 50 ng/mL), and NT-3 (to 1 ng/mL), and plated the cells in a 24-well plate on the coverslips coated with PDL and laminin initially.

DRG explants were cultivated and purified similarly; it is not necessary to cut up the cells before cell digestion and no filter was needed. The digestion time was shortened to 5 minutes.

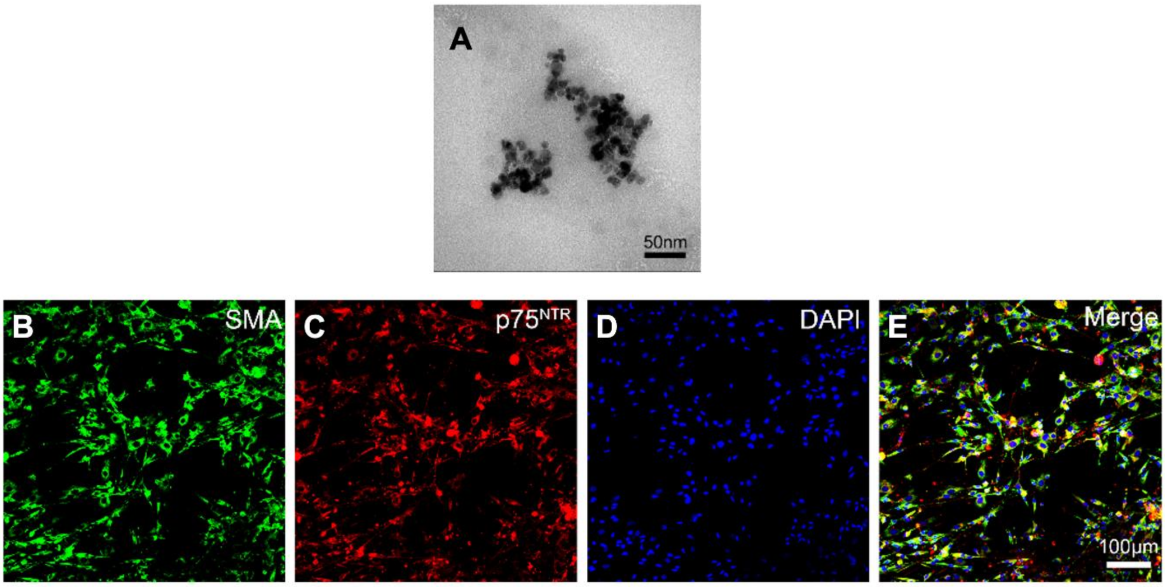

Figure S1. SPIONs and cell identification. (A)Representative TEM of the SPIONs. Primary cultures of OECs stained for SMA (B), p75^{NTR} (C), nuclei with DAPI (D), and merge files showed a purity of >95% OECs (E).

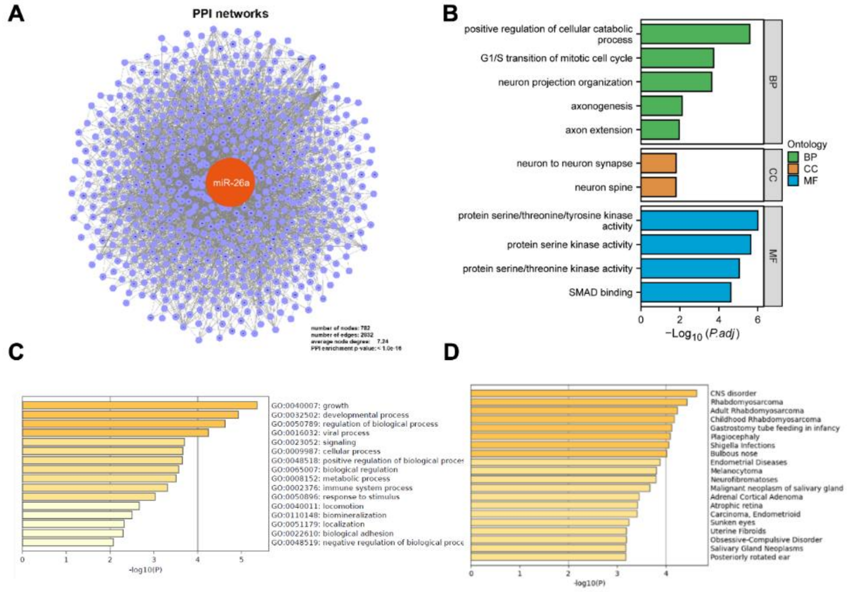

Figure S2. Functional enrichment of miR-26a and analysis of related genes. (A) The PPI networks of miR-26a. (B) Biological process terms from GO enrichment and KEGG pathway analyses of cluster III of the dysregulated proteins in sepsis. (C-D) The most significant items in miR-26a-related targets are classified according to the p-value.

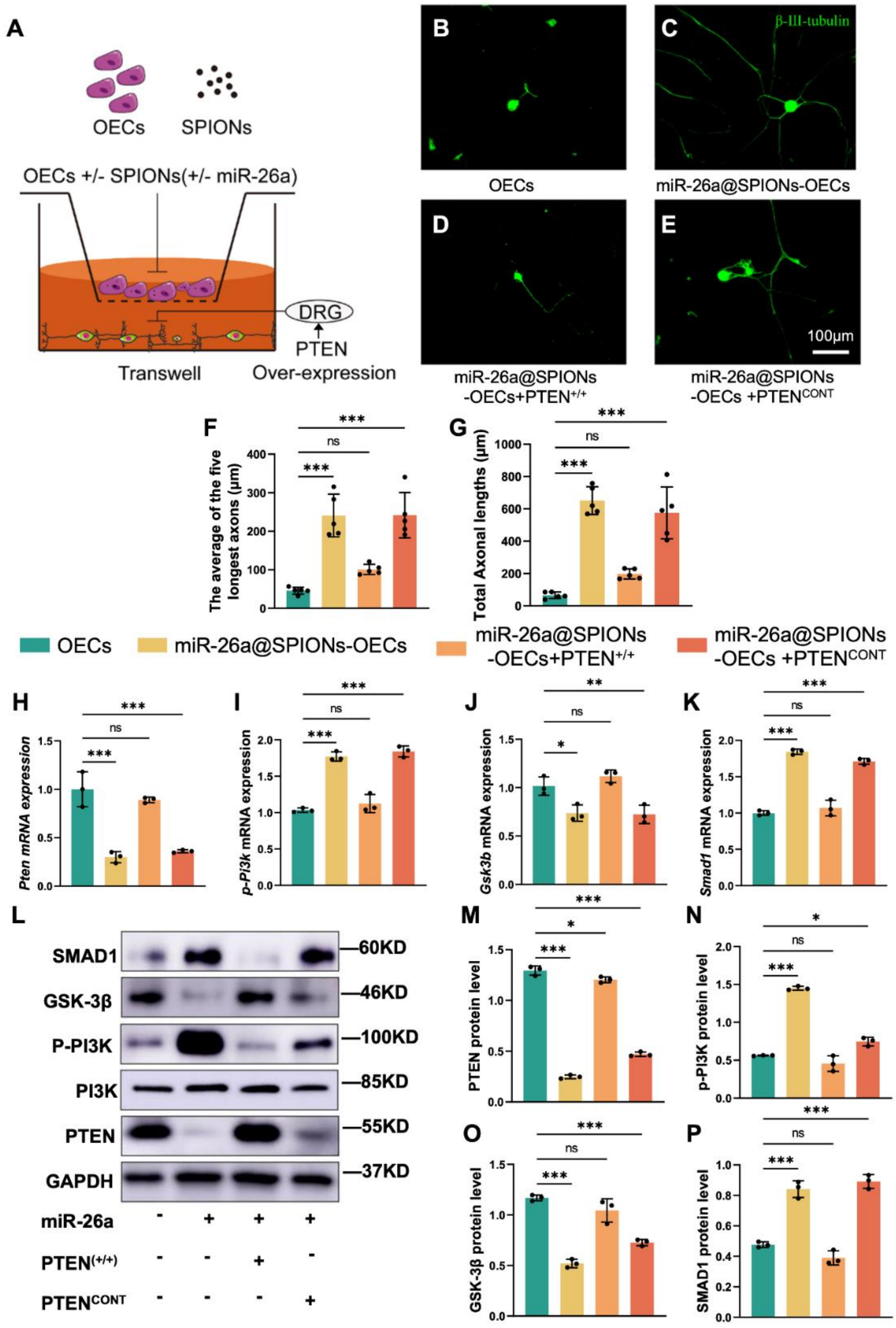

Figure S3. PTEN overexpression affects the effect of miR-26a on axon growth. (A) Schematic diagram of OECs co-cultured with PTEN overexpressed DRG neurons. (B-E) Axonal elongation of DRGs which co-cultured with OECs in different treatment groups after 2 days. Representative images of DRG neurons were stained for β-III-tubulin (green). (F-G) Axonal lengths were quantified and plotted for each treatment of three independent experiments. (n = 3, ****P* < 0.001 for comparison with OECs without treatment.) (H) *Pten*, (I) *p-Pi3k*, (J) *Gsk3b*, and (K)*Smad1* mRNA expression in different treatment DRGs after 2 days. (n = 3, **P* < 0.05, ***P* < 0.01 and ****P* < 0.001 for comparison with OECs without treatment.) (L) Detection of GAPDH, PTEN, P-PI3K, GSK-3β, SMAD1 protein expression in CONT, miR-26a@SPIONs and miR-26a@SPIONs+*Pten*$^{+/+}$, miR-26a@SPIONs+*Pten*^{CONT} by Western blotting. (M-P) Quantitative statistics of

WB results are in Figure L. (n = 3, *P < 0.05 and ***P < 0.001 for comparison with OECs without treatment.) All statistical data are analyzed by one-way ANOVA with Dunnett's multiple comparisons test and represented as mean ± SD, ns = no significance.

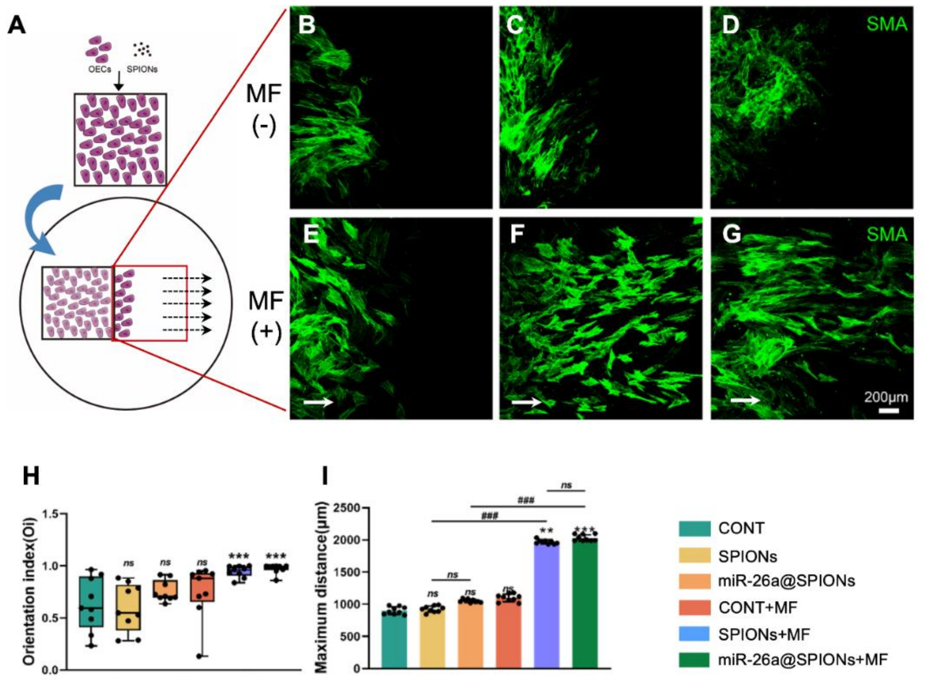

Figure S4. Growth and orientation of OECs in different treatment groups. (A) Schematic diagram of OECs slide inversion method. (B-G) Immunofluorescence staining of OECs in different treatment groups showed that OECs specifically expressed SMA (green). (H)OECs migration orientation index. The box plot showed the median, interquartile ranges, maximum, and minimum. (n = 9, **P < 0.01 and ***P < 0.001 for comparison with OECs without treatment.) (I) Average maximum distances of migration from the edge of the inverted fragments. (n = 9, **P < 0.01 and ***P < 0.001 for comparison with OECs without treatment; ###P < 0.001 for comparison with miR-26a@SPIONs-OECs+MF.) All statistical data are analyzed by one-way ANOVA with Dunnett's multiple comparisons test and represented as mean ± SD, CONT = the control group, ns = no significance.

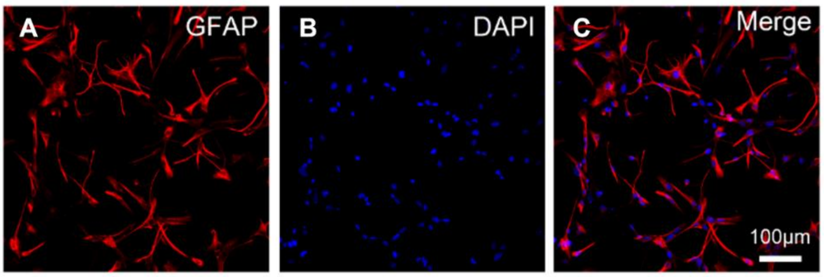

Figure S5. Astrocytes identification. Primary cultures of astrocytes stained for GFAP (A), nuclei with DAPI (B), and merge files (C) showed a purity of >90% astrocytes.

Supplementary Tables:

Table S1: Primer sequences used for PCR analyses in this study.

Gene	Primer Sequences

GAPDH-F	5'-GAAGCTGGTCATCAACGGGA-3'
GAPDH -R	5'-TCACCCCACTTGATGTTGGC-3'
Pten-F	5'-TCCCAGTCAGAGGCGCTATGTAT-3'
Pten -R	5'-GATGCTACCACACACAGGCAATG-3'
miR-26a-F	5'-GCCGTGGCCTTGTTCAAGTA-3'
miR-26a-R	5'-CCCCGTGCAAGTAACCAAGA-3'
Gfap-F	5'-CCTTGAGTCCTTGCGCGGC-3'
Gfap-R	5'-TTGGCCCTCCTCCTCCAGC-3'
PI3K-F:	5'-GCCCAGGCTTACTACAGAC-3'
PI3K-R	5'-AAGTAGGGAGGCATCTCG-3'
Gsk3b-F	5'-CGGGACCCAAATGTCAAACT-3'
Gsk3b-R	5'- CGTGACCAGTGTTGCTGAGT-3'
Smad1-F	5'- TCATCCCGGGAGGTGGCAGA-3'
Smad1-R	5'- GACCTCCTTCAGCCGCTGGT-3'

Table S2: miR-26a probe sequences in this study.

**Gene**	Primer Sequences
rno-miR-26a probe	AGCCUAUCCUGGAUUAUUGAA

Reference

[1] C. Radtke, J. D. Kocsis, Cells, tissues, organs 2014, 200 (1), 48,

[2] B. Xia, J. Gao, S. Li, L. Huang, T. Ma, L. Zhao, Y. Yang, J. Huang, Z. Luo, Front Cell Neurosci 2019, 13, 548,

[3] H. Shen, M. Gan, H. Yang, J. Zou, The Journal of international medical research 2019, 47 (7), 3253,

[4] J. B. Zuchero, Cold Spring Harbor protocols 2014, 2014 (8), 813.
